# Deeper insights into long-term survival heterogeneity of pancreatic ductal adenocarcinoma (PDAC) patients using integrative individual- and group-level transcriptome network analyses

**DOI:** 10.1038/s41598-022-14592-1

**Published:** 2022-06-30

**Authors:** Archana Bhardwaj, Claire Josse, Daniel Van Daele, Christophe Poulet, Marcela Chavez, Ingrid Struman, Kristel Van Steen

**Affiliations:** 1 GIGA-R Centre, BIO3 – Medical Genomics, University of Liège, Avenue de L’Hôpital, 11, 4000 Liège, Belgium; 2 Laboratory of Human Genetics, GIGA Research, University Hospital (CHU), Liège, Belgium; 3 Medical Oncology Department, CHU Liège, Liège, Belgium; 4 Department of Gastro-Enterology, University Hospital (CHU), Liège, Belgium; 5 Department of Medicine, Division of Hematology, University Hospital (CHU), Liège, Belgium; 6 GIGA-R Centre, Laboratory of Molecular Angiogenesis, University of Liège, Liège, Belgium; 7 Laboratory of Rheumatology, GIGA-R, University Hospital (CHULiege), Liège, Belgium

**Keywords:** Cancer, Computational biology and bioinformatics

## Abstract

Pancreatic ductal adenocarcinoma (PDAC) is categorized as the leading cause of cancer mortality worldwide. However, its predictive markers for long-term survival are not well known. It is interesting to delineate individual-specific perturbed genes when comparing long-term (LT) and short-term (ST) PDAC survivors and integrate individual- and group-based transcriptome profiling. Using a discovery cohort of 19 PDAC patients from CHU-Liège (Belgium), we first performed differential gene expression analysis comparing LT to ST survivor. Second, we adopted systems biology approaches to obtain clinically relevant gene modules. Third, we created individual-specific perturbation profiles. Furthermore, we used Degree-Aware disease gene prioritizing (DADA) method to develop PDAC disease modules; Network-based Integration of Multi-omics Data (NetICS) to integrate group-based and individual-specific perturbed genes in relation to PDAC LT survival. We identified 173 differentially expressed genes (DEGs) in ST and LT survivors and five modules (including 38 DEGs) showing associations to clinical traits. Validation of DEGs in the molecular lab suggested a role of REG4 and TSPAN8 in PDAC survival. Via NetICS and DADA, we identified various known oncogenes such as CUL1 and TGFB1. Our proposed analytic workflow shows the advantages of combining clinical and omics data as well as individual- and group-level transcriptome profiling.

## Introduction

Pancreatic ductal adenocarcinoma (PDAC) accounts for 90% of pancreatic tumors and 10% of gastrointestinal cancers^1^. It is the 4th leading cause of cancer-related death worldwide while remaining the most lethal among digestive cancers^2^ with only few treatment therapies^3,4^. PDAC has a complex and dense tumor microenvironment that poses a significant barrier to treatment administration^5^**.** In general**,** various factors shape the outcome for complex diseases leading to perturbations of a complex intracellular network^6^. Disease-relevant genes typically do not operate on their own^7^. Network approaches that naturally acknowledge interactions and allow integration with regulatory factors are thus required to map phenotypic variability of complex diseases, including PDAC fully.

For PDAC, various studies have shown the influence of lymph node, lymphovascular and perineural invasion, surgical resection margin, chemotherapy^8–10^ on prognosis. The overall survival of patients may also be coupled to the mutational status of Kirsten rat sarcoma viral oncogene (*KRAS*) as well as several morphological features^11^. Also, multiple miRNAs and transcription factors influence metastasis and overall survival time of PDAC patients^12,13^. Extensive and comprehensive genomic profiling of different cancer types using next-generation sequencing has already increased our insights into cancer pathologies to provide potential therapeutic routes^14–16^. Also, for PDAC, several studies exist that focused on the use of microarray^17,18^ and single-cell RNAseq^19^ towards revealing promising therapeutic targets. Due to the high lethality of PDAC, intensive research is needed to understand biological mechanisms and to further unravel roots of causes for PDAC survival in general and long-term (LT) versus short-term (ST) survival in particular. In the literature, several criteria for LT and ST survival exist. For instance, Duconseil and co-authors considered ST (resp. LT) survival as surviving ≤ 8 (resp. ≥ 8) months. They identified significant factors involved in PDAC progression, yet only considering clinical data^20^. Stark et al.^14,21^ focused on LT survival defined as ≥ 10 years of survival and used logistic regressions to predict LT survival via clinical data and tumor characteristics. Chen and colleagues explored the molecular characteristics of ST (<14 months) and very long-term survival (≥10 years) of survival using proteomics data^22^. Very little information is available about potential regulatory mechanisms involved in the context of < 12 months and ≥ 36 months of survival within European populations. We aim to fill this gap and to explore PDAC survival mechanisms by making use of genomics data and by integrating a variety of gene prioritization methods.

Multiple questions are of interest, including (a) ‘How do LT and ST PDAC survivors differ from each other using RNA-seq data resource’ and (b) ‘Which survival group is most heterogeneous in terms transcriptome signatures’. In order to address both the question, there is need to apply various promising tools to dissect patients specific gene expression profile. PDAC is the most common type of pancreatic cancer featured with intra-tumoral heterogeneity^19^. Indeed, heterogeneity poses a significant challenge to personalized treatments for PDAC^23^. Gene expression data is often used to identify differentially expressed genes (DEGs) between groups of interest^24^. Previous classification studies paved the path to a better classification of patients with PDAC. For example, Puleo and colleagues defined five PDAC subtypes based on features of cancer cells and the tumor microenvironment, showing associations with patient outcomes^25^. Bailey and colleagues pioneered the identification of subgroups of PDAC patients by using the information on molecular pathology^26^. Peran et al.^27^ classified TCGA PDAC patients by specific cancer-related molecular features to predict PDAC progression. The identification of subgroups by looking into a perturbed profile of each individual might be another interesting approach. Typically, such (molecular) subtyping analyses require relatively large sample sizes. Alternative and more elaborate approaches are required, better exploiting and combining individual-level and group level profiling, to address the aforementioned questions.

Pathological findings with tumor cells suggest an abundance of different gene regulatory networks in humans for various cancers including, breast^24,25^, prostate^30^, and PDAC cancer^31^. Network-based approaches to complex diseases^6^ are progressively being integrated into analysis workflows and allow the knowledge integration of molecular interactions. As such, network biology approaches can identify key regulators responsible for molecular heterogeneity, giving rise to LT and ST PDAC survivor subgroups. Weighted Gene Co-Expression Network Analysis (WGCNA) is such an approach and enables the identification of gene modules and their associations with phenotype-related measurements^32^, such as tumor size or other clinical features. More work is needed to explore the link among the individual based differential expressed genes to the clinical features.

In response to the above, we embarked on a pilot study to tease out PDAC survival associated genes, with a particular interest in LT survivors (≥ 36 months survival; in contrast to ST survival defined as ≤ 12 months survival) and individual-to-individual differences (PEEP: individual perturbation expression profiles) in whole transcriptome profiles. To this end, we introduced and implemented a flexible and interpretable omics integrative analysis framework involving a series of group-level and individual-level viewpoints. By the use of bioinformatics based multiple softwares, we identified group- and individual-level differential expressed genes and found their association with clinical features and PDAC specific disease module. We validated the differential expessed genes identified among ST and LT group in molecular lab confirmed a role of multiple genes in PDAC survival. We hope that the gene targets (group- and individual-level) identified based on our integrative analytical framework may potentially be useful for the individual assessment of each patient, which can eventually lead to the precision medicine.

## Results

### Patients characteristics

**Figure 1:**
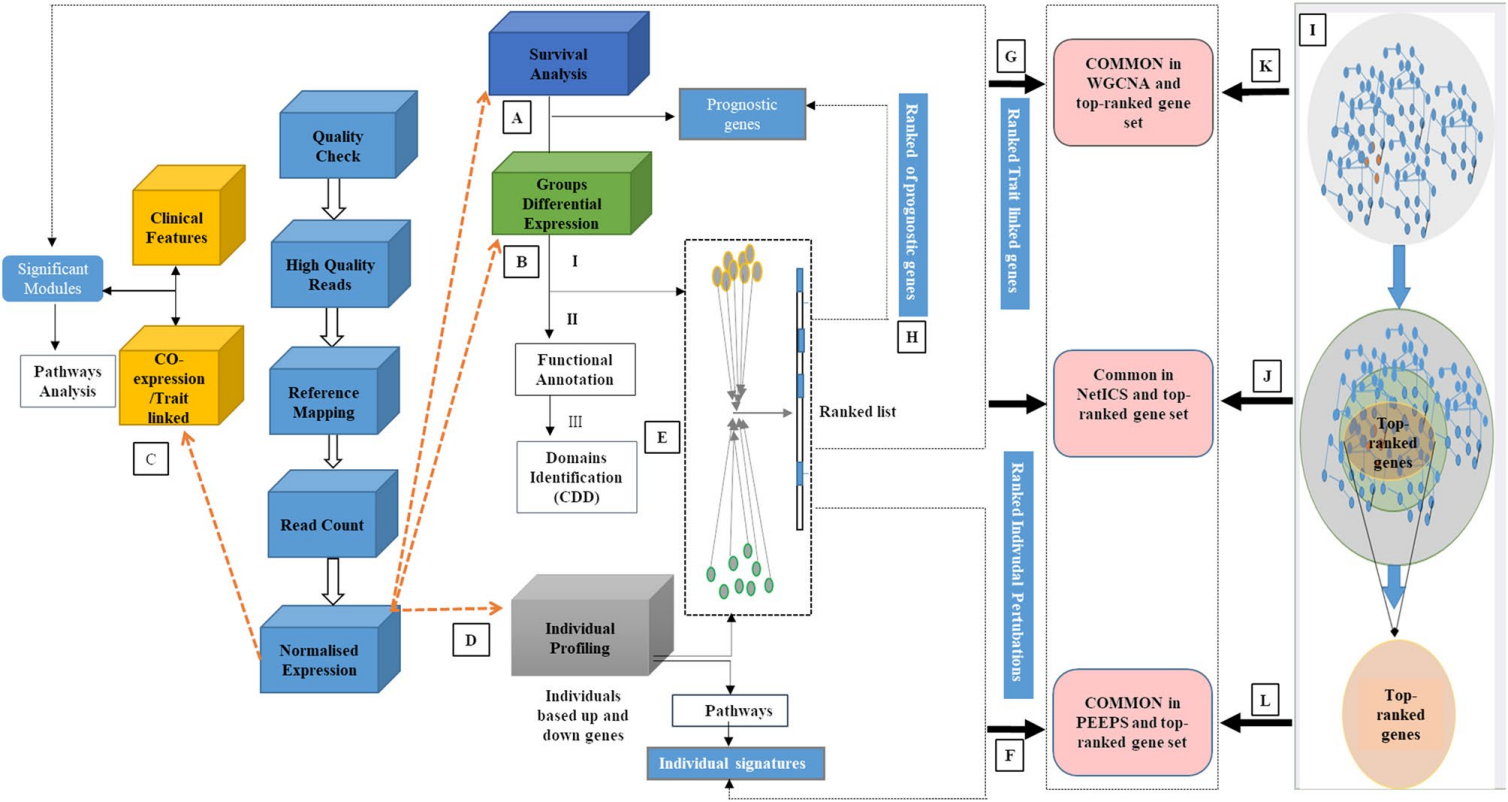
Flexible and interpretable omics integrative framework for RNA-seq data collected on two groups of patients, exemplified on PDAC ST/LT survival. RNA-seq quality-controlled data are inputted for (**A**) Survival analysis; (**B**) Group-based differential analysis via DESeq2^78^ (**C**) Weighted gene co-expression network analysis WGCNA^32^; (**D**) Individual-based differential analysis; (**E**) Genes are ranked based on the integration of individual and group-based differentially expressed genes via NetICS^92^; (**F**–**H**) NetICS specific top 1% ranked genes are traced back in multiple previous analyses (**A** through **E**); (**I**) DADA^7^ analysis starting from disease genes; (**J**–**L**) DADA specific top 1% ranked genes are traced back in previous analyses (**A** through **E**).

**Figure 2:**
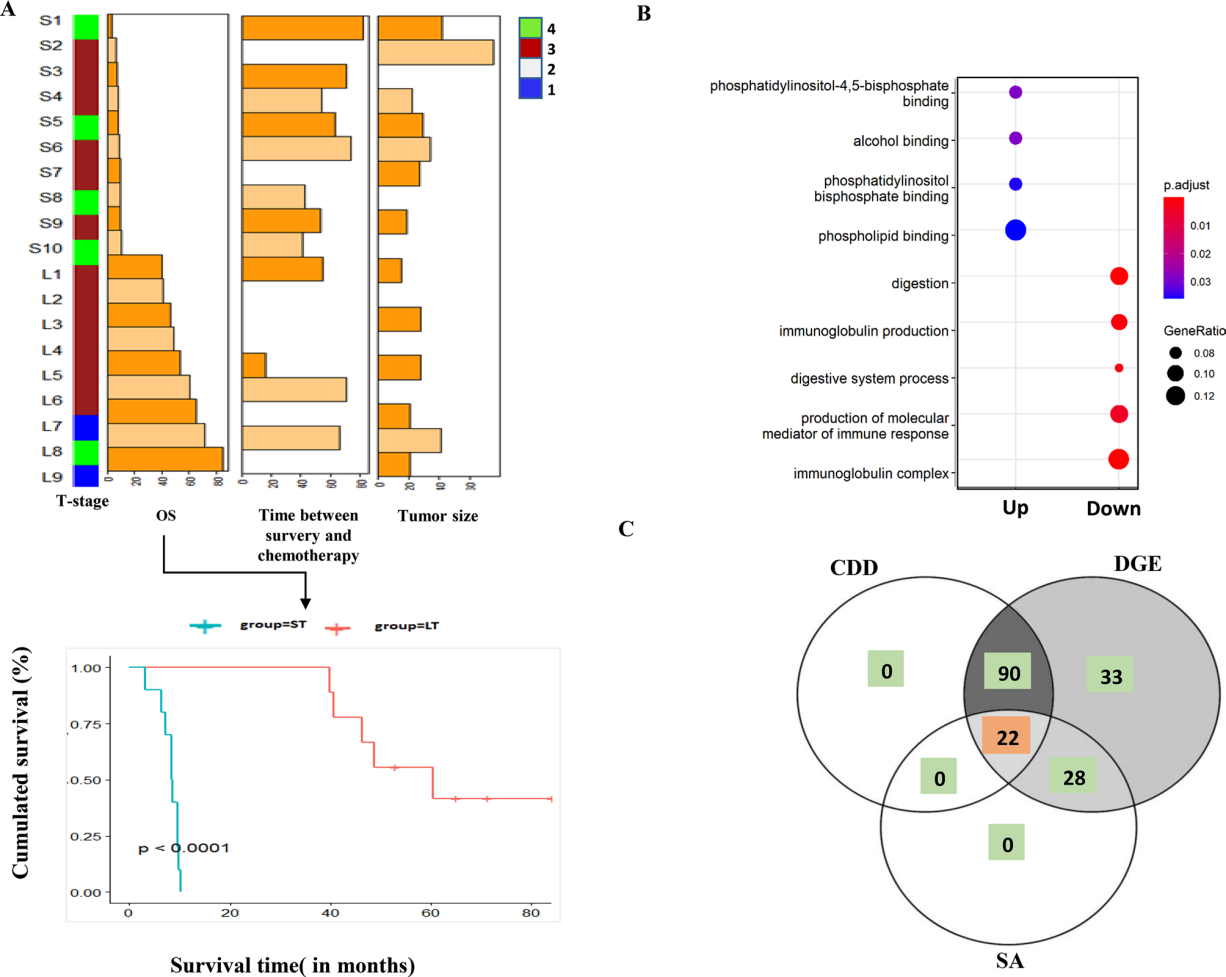
Overall Kaplan–Meier survival analysis of the ST and LT PDAC cohorts: **(A)** Patient characteristic data for a selection of PDAC relevant traits are shown as mixed bar and heat map plot. P1 to P13 refer to patient specific clinical traits analyzed in this study (selective data has been shown in plot; full details given in Table S1). Tumor stage (from 1 to 4). OS (in months), tumor size by imagery (in mm) and Time between imagery and surgery are indicated in the graph. OS clinical trait denotes overall survival and was used for the development of the Kaplan–Meier survival curves for short-term (ST) and long-term (LT) PDAC Survivors (ST: S1 to S10; LT: L1 to L9); (**B**) Identification of significant gene ontology of associated up and down-regulated DEGs and their relevant functions. Up and down-regulated genes are highlighted with red and green dots, respectively. The size of data points increases with increased significance (uncorrected for multiple testing); (**C**) Venn-diagram showing the number of identified genes that are common to or different in multiple first-line analysis strategies (CDD: conserved domain analysis, DGE: differential gene expression analysis, SA: survival analysis.

All patients were divided into ST (≤12 months) and LT (≥36 months) survival groups (resp. ST and LT), as summarized in Figs. 1 and 2A. Multiple bioinformatics methods were used for the biomarker identification at group and individual level (Fig. S1A). Detailed information about patient selection, ethical statement, and definition of ST and LT survivors is given in methods section. A total of 19 patients, comprising 10 ST and 9 LT, met our inclusion criteria. A complete list of some of the clinical features of ST and LT patients is given in Table S1.

### Differential gene expression analysis and functional follow-up

RNA was extracted from FFPE tissues, and a quality check was performed for paired-end sequencing (refers to methods section—RNA extraction, library preparation, sequencing). The long non-coding gene *MIR205HG* was the topmost differentially down-regulated gene in the LT group (*p*-value = 0.008). In contrast, the protein coding gene *GKN1,* which encodes for gastrokine1, was the topmost differential up-regulated gene in LT (*p*-value = 1.25E−05). Digestive system, immunoglobulin complex, immunoglobulin production specific gene ontology terms were uniquely enriched in down regulated genes while phospholipid binding specific GO terms were uniquely enriched for up-regulated genes (Fig. 2B; Table S2 and S3). A primary goal of molecular biology is to determine the mechanisms that regulate the transcription. Specific domain structures of genes play a significant role in gene regulation and expression. The conserved domain analysis resulted in 112 genes containing at least one domain (Fig. S1B; refers to methods section—Group based DEGs analysis). Sixteen genes contained an Ig domain, followed by a V-set domain. Based on the clusterprofiler based enrichment analysis of DEGs against the interpro domains, we identified three significant enrchied domains (Cytosolic fatty-acid binding, Intracellular lipid binding protein and Glycoprotein hormone subunit beta) under threshold of *p*.adjusted < 0.05.

Fifty three prognostic genes (*p*-value < 0.05) were identified from all DEGs (Table S4). We observed 22 DEG genes containing at least one domain that overlapped with the survival gene set (Fig. 2C). *GKN1* was found as part of oncogenic signatures (ATM_DN.V1_UP: c6 MSigDB dataset). Also, *GKN1* consisted of BRICHOS domain, found in a variety of proteins. We furthermore identified two genes *HIST1H1T,* and *SOX10* (disease-associated), consisting of the linker histone protein domain and Sox_HMG box, respectively, probably implying these genes’ regulatory role in PDAC survival mechanisms. Another gene, *miR-765,* showed a significant increase in survival in long-term patients with lower expression compared to ST (Fig. S2).These results highlight the potential of the identified genes in further understanding molecular underpinnings of PDAC survival. RT-qPCR confirmed the differential expression observed in LT versus ST for the genes represented in Fig. S2A. Among them, the DEGs *REG4* and *TSPAN8* were validated in the lab via RT-qPCR analysis (Fig. S3**).** In addition, several DEGs from studied cohort such as *CRISP3, PCK1, TRIM31, GPR87, SOWAHA, CITED1, NUTM2A, HAVCR1, ANXA8, PMP2, CXCL17, SCGB3A1* were identified in collision et al. 2016 cohort as well. Similarly, few more genes from Notta et al. 2016 cohort (9: *[KRT6A, IGKV1D-33, IGKV1-39, IGKV1-8, IGHV3-43, HP, ANXA8, IGKV1-6, CYP27C1])* and TCGA cohort ([11: *(GPR87,PRSS41,OTC,KRT6A,TAC1,ANXA8,MUC16,LYPD2,PGC,DKK4,LYZ]),* respectively shows the overlapping with DEGs identified from the current study*.* Overlapped genes to multiple cohorts furthermore confirm their role in PDAC survival.

### Group-level survival heterogeneity: gene co-expression modules significantly associated with clinical traits and their corresponding 3D architectures

**Figure 3:**
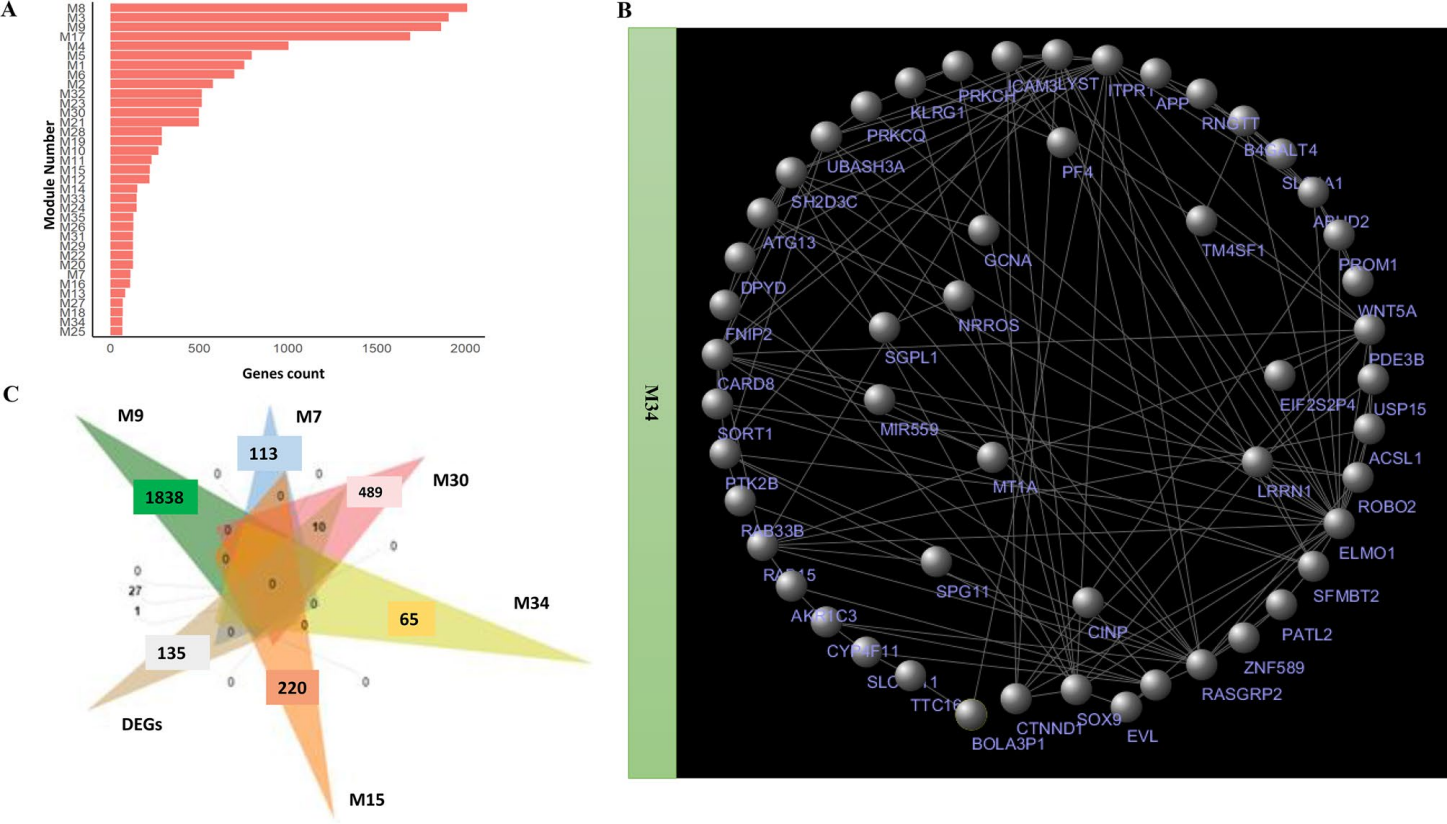
Clinical relevance of gene co-expression modules: (**A**) Bargraph indicating the number of genes involved in each WGCNA-derived gene module; (**B**) Network topology of one of the M34, where nodes are genes and connections among nodes represent gene–gene interactions. In each network, the gene names are indicated in the circular layout as derived from Cytoscape^86^. (**C**) Venn diagram indicating the common genes between the identified significant DEGs and the five previously identified clinically relevant modules.

All 19 samples with clinical information and gene expression data were included in WGCNA (refers to methods section—Group-level survival heterogeneity)*.* Genes with similar expressions were grouped into gene modules via average linkage hierarchal clustering. In this study, power of β = 14 (scale free r^2^ > 0.85) was selected as soft thresholding to ensure the scale free topology. By use of a dynamic tree-cutting algorithm, a total of 96 distinct co-expression modules were identified. Correlated modules were merged with a cut-off height of 0.25, resulting in 35 modules containing 66 to 2010 genes per module. Module M34 was the smallest module consisting of 66 genes, whereas M8 was the largest module comprising 2010 genes (Figs. 1C and 3A). The identified 35 modules covered 97 percent of the 18,880 input genes. For those 35 modules, we derived the corresponding module eigengenes.

Association of clinical features with dysregulated genes may help to clarify which genes might be important for disease development. All identified DEGs (173 in total) were distributed in 25 modules. Five modules had a significant correlation with clinical phenotypes (with the threshold of Bonferroni multiple testing adjusted *p*-value < 0.05): M7, M9, M15, M30, and M34 (Fig. S4). Clinically relevant significant modules were imported into Cytoscape, and gene–gene interactome network were developed for M34 module (Fig. 3B). Module M9 was found to be significantly associated with tumor size (r^2^ = 0.72, adjusted *p*-value = 0.01) and T stage (r^2^ = 0.68, adjusted *p*-value = 0.03). M9 consisted of the highest number of DEGs (27 genes). Two other modules, M7 (r^2^ = 0.73, adjusted *p*-value = 0.01) and M30 (r^2^ = 0.71, adjusted *p*-value = 0.02), were negatively associated with time between surgery and chemotherapy clinical traits. M30 contained 10 DEGs. Module M34 was significantly associated with tumor size by imagery (r^2^ = 0.67, adjusted *p*-value < 0.05). Interestingly, two modules were significantly associated with chemotherapy: a positive association for M15 (r^2^ = 0.68, adjusted *p*-value = 0.04) and a negative association for M9 (r^2^ = −0.68, adjusted *p*-value < 0.04). The overlap between DEGs and genes in five modules (M7, M9, M15, M30, M34) is shown in a Venn-Diagram (Fig. 3C), from which we can identify 27, 10, and 1 gene as part of M9, M30, and M34, respectively.

### Group-level survival heterogeneity: functional analysis of clinically relevant gene co-expression modules

**Figure 4:**
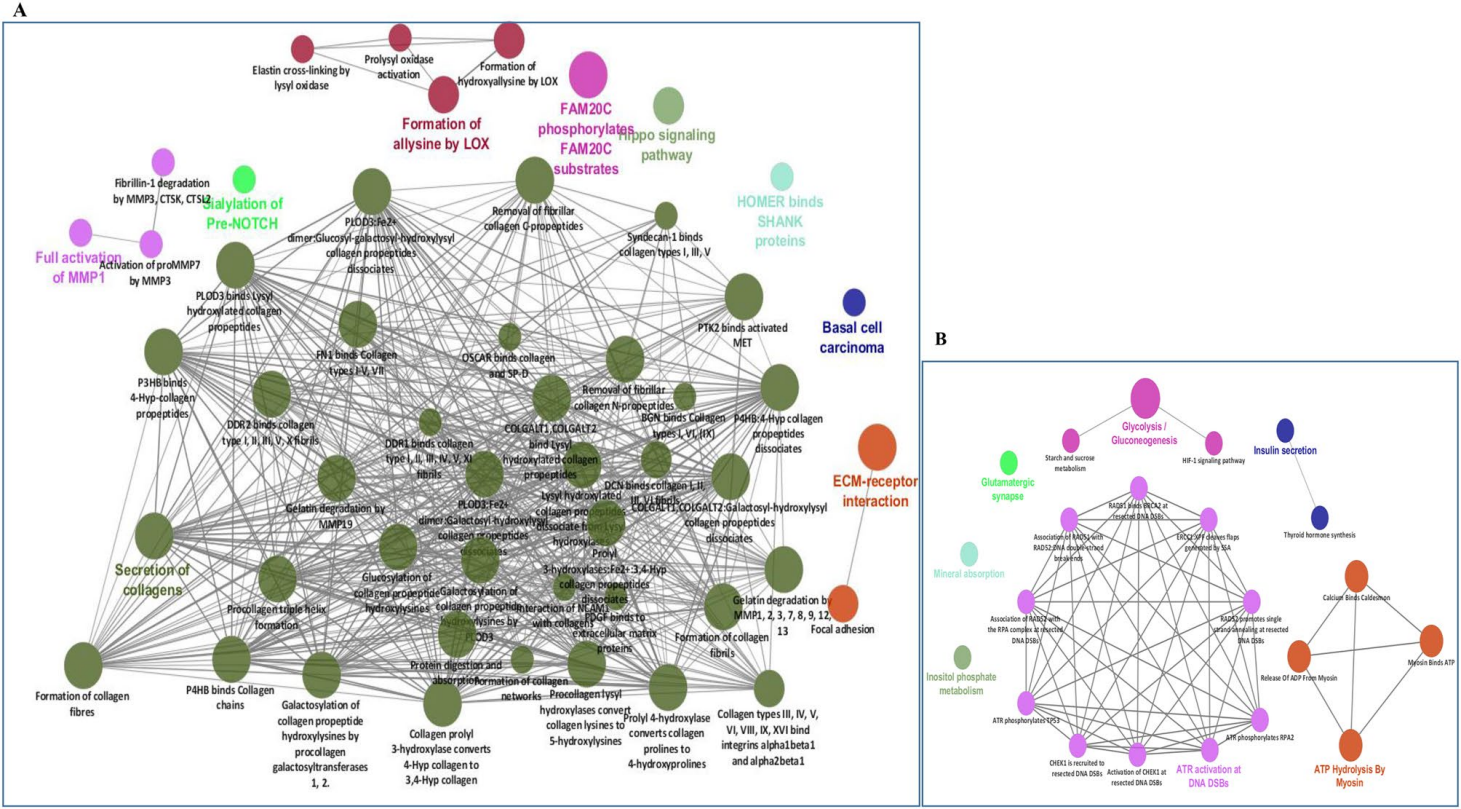
Functional follow-up of clinically relevant gene expression modules: (**A**) Ten groups for module M9 comprising 33 significantly linked pathways; (**B**) Depiction of the five groups identified in M15; For A-B, redundant groups with > 50% overlap were merged. Each node in the network represents an enriched term; the size of each node follows the extent of enrichment significance. Connection among different nodes are based on kappa scores (≥ 0.4), as available from ClueGO.

Clinically relevant gene modules (i.e., modules identified by WGCNA as significantly associated with clinical traits) were functionally followed up in Cytoscape with the ClueGO plug-in (Group-level and Individual-specific analyses) that visualizes large clusters of genes in a functionally grouped network. Module M9 was linked to 33 significant pathways (multiple testing adjusted *p*-value < 0.05) distributed over ten groups, such as extracellular matrix organization (86 genes) and collagen formation (37 genes) (data not shown). Genes regulating the cell cycle and modulating extracellular matrix at molecular or cellular levels have been linked to cancer drug targeting and cancer cell plasticity^32^. M7, also negatively associated with chemotherapy, contained 91 significant pathways, distributed into three groups, such as proteasome (4 genes) and the regulation of *RAS* by *GAP*s (5 genes) (Fig. 4A). Module M15, positively associated with ‘chemotherapy’, was enriched with 11 significant pathways distributed into five groups, such as the inositol phosphate metabolism (3 genes) and muscle contraction (9 genes) (Fig. 4B). In module M34, we found three significant Reactome pathways distributed into three groups: the effects of PIP2 hydrolysis (4 genes), the deactivation of the beta-catenin transactivating complex (3 genes) and the VEGFA-VEGFR2 pathway (4 genes) (data not shown). In M30, we found two significant pathways: apoptotic cleavage of cell adhesion proteins (4 genes) and o-linked glycosylation (11 genes) (data not shown). Bailey et al.^26^ reported four subtypes in PDAC i.e. ADEX; Immunogenic; Squamous; Pancreatic Progenitor. Based on SubMap module based analysis in GenePattern (https://www.genepattern.org/), we found that ST and LT show significant association with Squamous (A3) and Immunogenic (A2) subtypes, respectively (Fig. S5) which indicates the role of immune system in the PDAC survival. Out of five subtypes from Puleo et al. 2018, 55% of the PDAC LT patients shows the significant association with immune and pure classical subtypes (Table S10). Both pure classical and immune classical subtypes known for good prognosis. Furthermore, enrichment of various immune specific pathways from clinical relevant modules signifies the potential role in PDAC survival.

### Individual-specific survival heterogeneity: quantification of heterogeneity between individual transcriptome profiles

**Figure 5:**
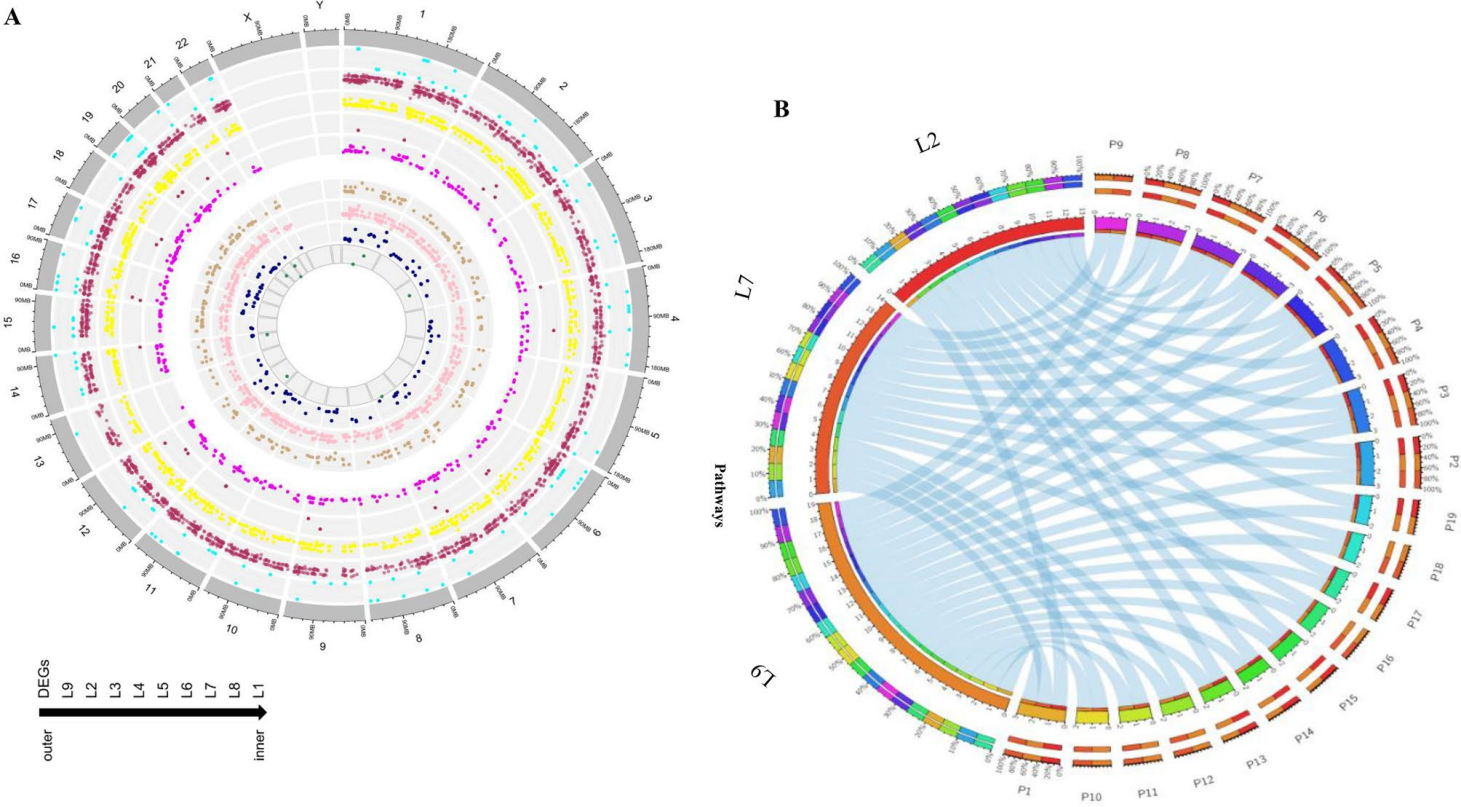
Genomic distributions of differentially expressed genes (DEGs) and PEEPs related to PDAC survivors using Circos plots and functional profiles of perturbation data: (**A**) first (grey) and second outermost circle labeled with numbers represent chromosomes (multiple colors); the third outermost track represents DEGs (red and green indicating, respectively up-regulated and down-regulated DEGs as scattered points); the fourth outermost circle represents genomic locations of genes associated with survival (purple lines); the nine innermost circles (highlighted in orange) refer to the z-score for each LT survivor (ranging from LT1 to LT9); (**B**) Enriched KEGG pathways (P1 to P19 (out of 196)) shown via Circos Table Viewer. Each link refers to an LT survivor and a significantly enriched pathway (adjusted *p*-value < 0.05) based on the perturbed gene set found in that individual (data for LT2, LT7 and LT9 are shown). Uniquely enriched pathways across LT survivors are given in Table S2.

To assess heterogeneity in long-term survival patients, we constructed individual perturbation expression profiles (PEEPs)^24^ (refers to methods section—Individual-specific survival heterogeneity)*.* It resulted in 6336 significantly perturbed genes across LT PDAC survivors (Figs. 1D and 5A). The frequency of disrupted genes in each LT survivor Li (i = 1,…,9) was L1:12, L2:1412, L3:43, L4:474, L5:179, L6:319, L7:957, L8:150 and L9:2789 (Fig. 5A). Various genes were uniquely perturbed in one LT patient only. Only a single group-wise DEG, out of 173 DEGs, was shared among 3 LT survival subjects, namely TNNI3. Also, at most six DEGs (IRS4, KLRC3, CLDN18, NPY, CNTN6, TAC1) were common to 2 out of 9 patients. Hence, for the majority of perturbed genes shared among LT survivors, no evidence was found about them being differentially expressed in a group comparison between LT and ST survivors. Among genes other than significant DEGs, only one was common to 7 out of 9 individuals: *NOSTRIN*, associated with nitric oxide pathways. No other genes were shared by 8 or all 9 LT. Five out of 9 LT patients shared *DTYMK* as a perturbed gene in their individual transcriptome profile or PEEP. Six genes (*PDXDC1*, *ATF7IP2*, *LIN7C JTB*, *TTL*, *DVL2*), which regulate the ERG signal transduction pathways, were retained in 4 out of 9 LT patients, were significantly involved in transcriptional mis-regulation in cancer (multiple testing adjusted *p*-value = 0.025). There were respectively 41 and 180 genes conserved in 3 and 2 out of 9 LT survivors. We also assessed the frequency of PEEPs (for individual based analysis) in LT survivors in three distinct cohorts. The result revealed a total of 278, 524 and 94 PEEPs that were depicted from independent cohort A, B and C, respectively. From cohort A, we found 3 genes perturbed in atleast 2 LT patient only. On the other hand, we observed 7 *(AF038458.4, COL26A1, CTD-2192J16.17, FLJ46284, GPR26, RP11-329N15.3, RP11-744H18.1)* genes that were perturbed in atleast 2 LT patients in cohort B. In cohort C, we found one of the *FXYD4* gene that perturbed in atleast 2 LT patients. Heatmap of identified PEEPs in three cohorts is given in Fig. S6. Individual level analysis in LT cohorts confirms the higher heterogeneity in LT survivors.

All perturbed gene sets are displayed in a circular plot (Fig. 5A). Two-way clustering (biclustering) of perturbed genes in PEEPs (gene is significantly perturbed or not) in LT highlighted 64 gene clusters (Fig. S7). The largest cluster (cluster 15) consisted of 363 genes. Deeper hierarchical clustering of previously identified clusters grouped cluster 7, 36,37,42,47,48,50,53,55 into a single supercluster (Fig. S7A) with over-representation of cancer-specific pathways such as mTOR pathways and NOD-like signalling pathways (Figs. S7B and S7C).

### Individual-specific survival heterogeneity: functional pathway and domain analysis in long-term PDAC survivors

We furthermore examined the extent to which the individual patterns in LT survivors reflected disruptions in KEGG and Reactome pathways and identified multiple pathways that were significantly enriched in at least one LT individual (Table S5). In-depth analysis revealed that 17 pathways (out of 192) were common to at least two LT survivors (Fig. 5B). Thus, 175 pathways were uniquely perturbed in an LT PDAC survivor (i.e. not shared among LT survivors). Individuals (LT1, LT3, LT4, LT5, LT6) did not show significant enrichment in any KEGG/Reactome pathway. Based on the presence/absence of enriched pathways across LT survivors (LT2, LT7, LT8, LT9), two-way hierarchical clustering revealed three clusters (Fig. S8). First two clusters (C1 and C2) showed enriched pathways in two LT only. C1 consisted of 14 pathways was collectively enriched in L7 and L9 and highlighted a strong association with cancer-related pathways. C2 showed enrichment of 13 pathways between L9 and L2, such as Proteoglycans in cancer and EPH-Ephrin signaling. Smallest cluster, C3, consisted of 8 pathways across three LT survivors, i.e. LT2, LT7, LT9**.** Deeper hierarchical clustering groups C2 and C3 into single supercluster based on similar pathways profiles.

A primary goal of molecular biology is to determine the mechanisms that control gene transcription. Specific domain structures of genes play a significant role in gene regulation and expression. Hence, we also investigated the domain structures of perturbed genes in PEEPs of LT to understand their potential regulatory mechanism in LT survival. A total of 47 enriched domains (adjusted *p*-value < 0.05) were identified (Table S6). Two-way hierarchical clustering (biclustering) based on motif enrichment profiles (present or absent) across all LT survivors resulted in four clusters (Fig. S8). The first cluster (C1), represented by LT7 and LT9, was enriched with six domains. The second cluster (C2), active in LT2 and LT7, was enriched with seven domains. The third clusters (C3) involved enrichment of 7 domains shared two among LT survivors (Table S6). The fourth cluster (C4) was largely shared by three LT survivors (LT2, LT7, and LT9). This cluster involved five domains: IPR013032, PS01186, IPR000742, PS00022, and IPR009030. Deeper hierarchical clustering groups C1 and C4 into single supercluster based on similar protein domains profiles.

In addition, for each LT survivor, we constructed two hierarchal trees based on the genes potentially involved in multiple domains and pathways, one for each for LT survivor. More in-depth analysis revealed a common gene set between cluster 24 obtained from gene-level clustering and cluster 1 (C1) derived from pathway-level biclustering (Figs. S8, S9 and S10). Similarly, cluster 25 derived from gene level analysis showed overlap with cluster 2 (C2) derived from pathway-level biclustering.

### Exploitation of gene connectivity: systems views

**Figure 6:**
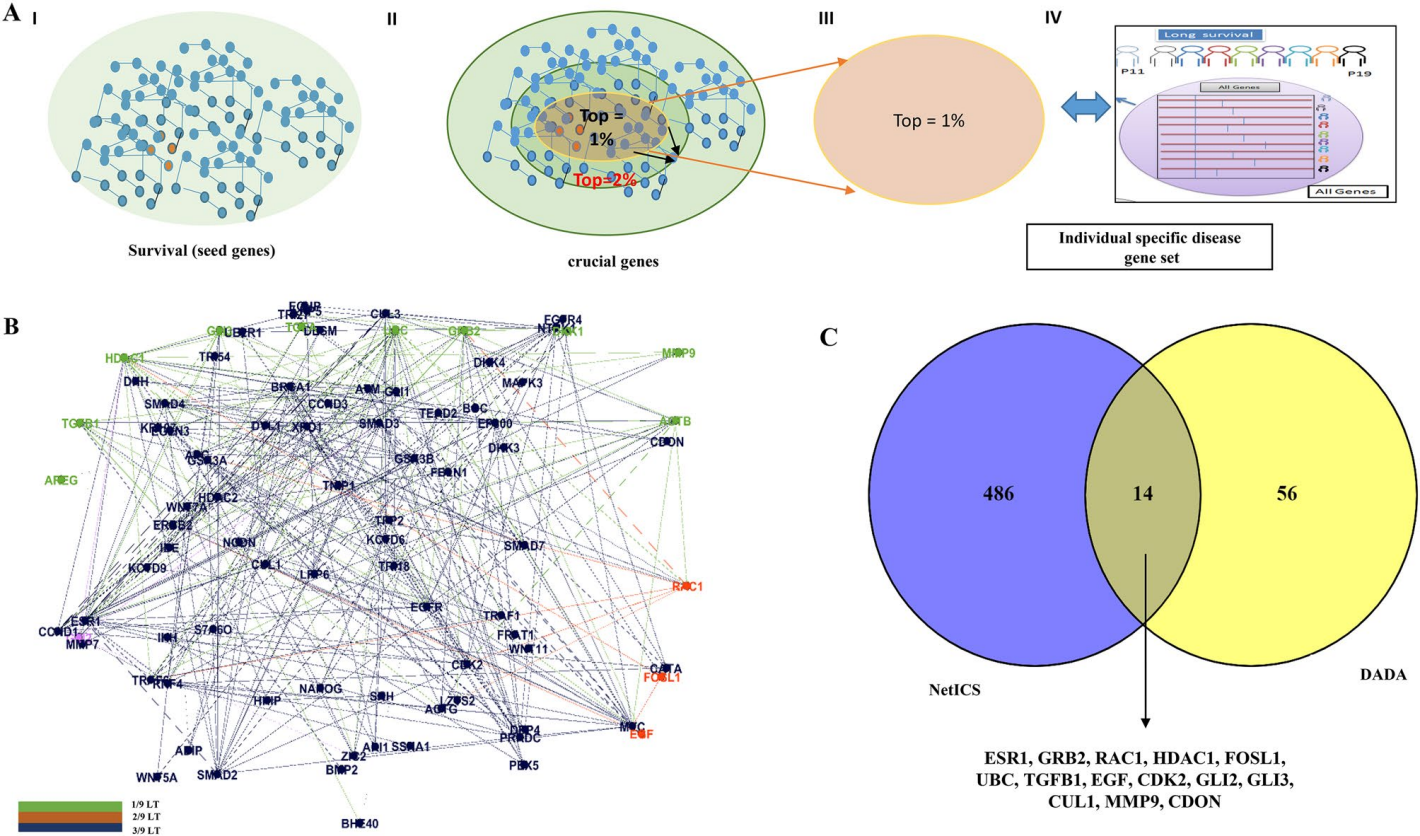
Exploitation of gene connectivity for LT PDAC survivor gene prioritization: (**A**) DADA-oriented multi-step disease module identification: PDAC seed gene selection (I), restriction to top 1% of ranked genes (II–III) and intersection of retained gene list with individual perturbation gene expression profiles for LT survivors (IV); (**B**) DADA-derived top-ranked genes found in at least one, two, or three LT survivors, indicated in green, orange and pink, respectively; (**C**) Common genes to DADA and other gene prioritization approaches: DEGs, clinically relevant WGCNA gene modules, and PEEPs;

Gene connectivity via reference networks can further highlight interesting gene clusters linked to LT survivors. In a first approach, we developed a disease module via DADA^7,33^. The latter uses the human protein interactome network structure to prioritize disease genes while also removing possible biases induced by gene degree distributions (refers to methods section—Individual-specific survival heterogeneity). The disease module hypothesis proposes that disease regulatory genes form one or a few large connected components in a human interactome. In this study, we restricted our seed genes (i.e., genes that play significant roles in PDAC according to the prior biological knowledge) to PDAC survival (*SMAD4*, *CDKA2*, and *KRAS*) and PDAC responsiveness based on a literature search and as identified from the DisGeNET database^34^ (Table S7). Only the top 1% of DADA ranked genes were retained (Figs. 6A I–IV; 1J), leading to 70 genes. Only one DADA top gene was also previously identified as DEG (*DKK4*), as shown in (Fig. 6C). We also looked at the overlap between DADA-based 1% top-ranked genes and perturbed genes as highlighted by the PEEPs of individuals belonging to the long-term survival PDAC patient group (Fig. 1L). There were 23 genes in total. None of these common genes had previously been identified as DEGs. Out of 23, we identified 7 DADA top-ranked genes in common to clinical gene modules as identified before (Fig. 6C, 1K; Table S6). Only a single gene was shared by at least (actually exactly) three LT subjects, namely *GLI2*. Three genes (*RAC1*, *FOSL1*, and *EGF*) were shared by two out of 9 LT survivor PEEPs. Furthermore, three genes (*JAG2*, *TGFA*, *HDAC1*) were uniquely perturbed in a LT survivor (Fig. 6B).

**Figure 7:**
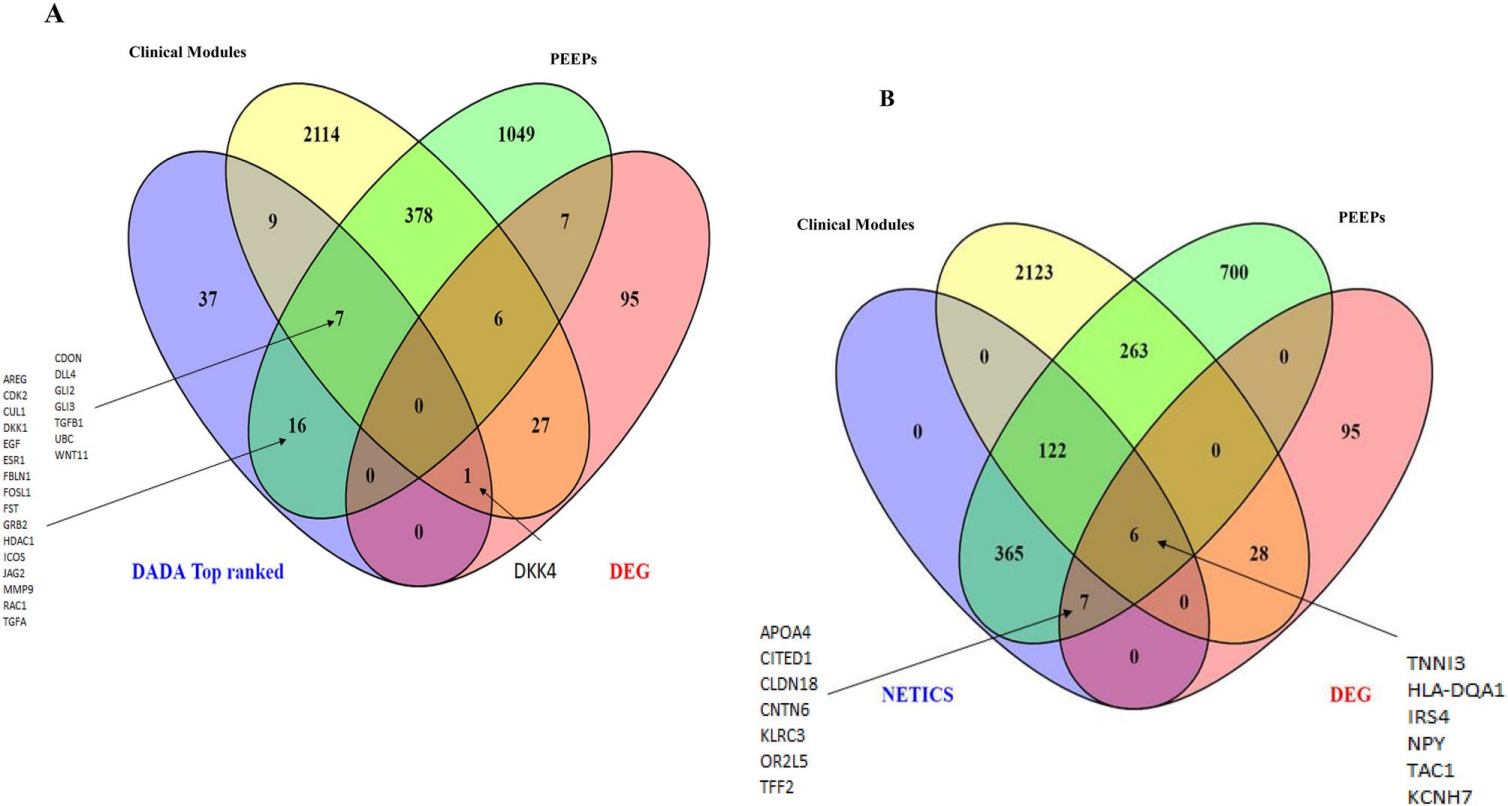
Common genes in multiple analysis: (**A**) Common genes to NetICS and other gene prioritization approaches: DEGs, clinically relevant WGCNA gene modules, and PEEPs; (**B**) Venn diagram showing the overlap between genes prioritized via NetICS and DADA. Common genes to top 1% NetICS individual gene lists and top 1% DADA genes are highlighted via arrows in Figs. 6C and 7A.

We integrated individual-specific gene perturbation information (from PEEPs) with group-level DEG findings. For this, we used NetICS, which further allows unraveling inter-and intra-patient gene expression heterogeneity (Individual-specific survival heterogeneity; Fig. 1E). Also, in this approach, a ranked list of genes was generated. The ranks are based on the gene scores acquired through network diffusion algorithms (Fig. 7A, Table S8). Similar to the DADA approach, we focused on the top 1% of ranked genes for each LT survival patient, leading to 500 genes. Those 500 genes constituted a subset of PEEP genes. Only 13 genes out of 500 were also DEGs, including 6 genes that were additionally linked to clinical disease modules (Fig. 7A). Among these 13 DEGs, *TNNI3* was NetICS top ranked, and was shared in its significance by 3 out of 9 LT survivors. It was also associated with the M7 module of clinical relevance (Figs. 7A, 1G; Table S8). Notably, *NOSTRIN*, a unique to NetICS gene (i.e., not highlighted by any other method shown in Fig. 7A) was common to 7 out of 9 LT subjects. Furthermore, we found 14 genes common to DADA and NetICS gene prioritization methodologies (Figs. 7B, 1J; S11; Table S9), involving the pathways such GPCR, Notch signaling pathway and many others. This common gene set did not include *TNNI3* nor *NOSTRIN*. The percentage of LT PEEP genes not included in the top 1% DADA gene list is 27% (384/1440) and is similar to the percentage of LT PEEP genes not included in the top 1% NetICS gene list (263/963).

## Discussion

Identifying molecular PDAC cancer drivers is critical for implementing precision medicine in clinical practice. Typically, the optimization and fine tuning of gene prioritization methods require large datasets^35^. Despite the small sample size of this study, we identified genes showing associations with multiple clinical traits^36^ and derived plausible links between long-term survival of patients and genes, pathways and protein domains by exploiting multiple approaches, including the combination of individual-level with group-level information in integrated analysis workflows. Throughout the entire study, we have relied on several statistical techniques and approaches to determine statistical significance with small samples (including non-parametric tests and empirically derived *p*-values).

PDAC accounts for over 90% of pancreatic cancer and is a lethal malignancy with very high mortality rates. The gene regulatory landscape of PDAC is defined by four mutational “mountains” (*KRAS*, *TP53*, *CDKN2A*, *SMAD4*), which are the main drivers of PDAC^37^. Thus, cancer diseases are heterogeneous at different scales: group level, individual level, tumor type, cell level. This study reports on PDAC gene expression differences in patients who survived longer than 36 months (LT) or less than 12 months (ST). Via advanced genomic profiling of PDAC survivors, we aimed to obtain more insights into LTS-relevant biological mechanisms that contribute to PDAC heterogeneity.

In this work, we identified known PDAC driver genes associated with survival, including *ROBO2, ZG16B*, ANXA8, CEACAM5, CYP24A1, GPR87, GSDMC, KLK6, KRT14, KRT6A, MMP13, MUC16, S100A2, SERPINB3, TRIM31, TSPAN8 and *PLXNA1*^38–40^ (Supplementary S2). Concordance result has been observed in the independent cohorts as well. In addition, a thorough investigation of gene expression differences between long-term and short-term PDAC survivors highlighted gene involvement in immune responses (*CEACAM20*, *C6orf13*, *IRS4*, *CXCL17*), cell cycle (*SPDYE3*, *HLA-DQA2*, *CLDN*) and metabolic pathways (*GBA3*, *LIPN*), further highlighting the importance of these pathways in PDAC disease sruvival^41,42^. Association of LT survivors with Immunogenic (A2) subtypes (Bailey et al. 2016) confirms the importance of identified immune specific pathways. These findings provide mounting evidence that differential expressed genes (*FABP2 , IGKV1D-8, TFF1, TFF2*) linked to immune responses could be useful in the development of effective therapies for PDAC survival^43^. Subtypes analysis based on two different studies (Puleo et al. 2018 and Bailey et al. 2016) ensures the role of immune genic pathways in good prognosis in LT PDAC survvuors in PDAC.

We also identified a downstream target of *KRAS* (*MUC16*) as DEG, supporting *KRAS* implications in survival^44^. Also, we observed modifications of *GKN1*, *KRT6*, and *ANKRD43* gene expressions in LTS, known to induce apoptosis and a higher metastasis in other cancer type^45,46^. In addition, a previous study showed *REG4* as a serological marker for PDAC^47^. Very little information exists, though, about the role of *TSPAN8* in PDAC. However, TSPAN8 was recently shown to promote cancer cell stemness via activation of sonic Hedgehog signaling^48^. Validation of a selection of DEGs via experimental work confirmed a role of *REG4* and *TSPAN8* in PDAC survival mechanisms. These molecular lab results indicate the interplay between the procession of tumorigenesis in PDAC and whole-body metabolism^49^, which could be regulated individually or in combination with various factors in survival patients. The presence of multiple immunogenic domains (IGV, V-SET) in identified DEGs further supports recent activities towards immunological targets for cancer therapy^50^. This indicates in-depth investigation of immunity cycles in relation to long-term survival in PDAC patients.

Systems biology approaches can provide immediate functional insights by revealing interactions between genes and cross-talks between biological processes^51^. A motivation for WGCNA is that genes functioning together are regulated or co-expressed together^52^. Ballouz and cauthor^53^ suggested a minimal of 20 samples to predict meaningful functional connectivity. This forced us to pool ST and LT together for WGCNA analysis on 19 patients and to link thus identified gene modules or their constituents to clinical traits with non-parametric statistics whenever appropriate. Multiple studies have indicated an association of early survival in PDAC to tumor size^54^.

Additionally, multiple targets have been identified in the form of DEGs being associated with numerous traits such as tumor size and the time between surgery and chemotherapy. In our study, we identified several clinically relevant WGCNA gene modules (e.g., a gene module associated to time between surgery and chemotherapy with DEGs *LYZ, DKK4, CA14, NASE7, TSPAN8, GKN1, GKN2, SNORD116-18, DKK4*), which warrants further exploration on increased sample sizes in the future. Notably, *TSPAN8* serves as a prognostic marker in other cancer types as well^48^. Apart from time between surgery and chemotherapy, time to surgery may play an important role in PDAC that has been associated with an increase in tumor size^55^. DEG *DKK4* (also top 1% DADA gene) is the least studied protein from the Dickkopf (*DKK*) family, which includes *DKK3*^56^ and *DKK1*^56^. The fact that *DKK4* did not appear in NetICS’s prioritization gene list, nor in PEEPs of LTS, suggests that DKK4 may be more promising in controlling the survival of patients with PDAC rather than explaining individual heterogeneity among long-term PDAC survivors. Identfication of DKK4 as group based DEGs in TCGA cohort further confirm its role in PDAC survival.

The identification of prognostic factors is complicated in the presence of individual-to-individual heterogeneity^57^. Unique tumour biology may determine long-term survival in pancreatic cancer, and detailed individual-specific omics profiling may be required to provide novel insights into prognostication for this disease^58^. DEGs alone are unlikely to fully characterize individual (LT) survival, as observed for other complex traits^24^. Previous studies^26,26,59,60^ emphasized the existence of subgrouping of PDAC patients in general, based on expression profiling of samples. Our study showed that any LTS patient only exhibits a small fraction of group-wise DEGs in their PEEP profiles and shows a deep level of gene expression heterogeneity. Notably, several genes were uniquely perturbed in an LT survivor, which strengthens our belief that LTS patients exhibit more abundant levels of heterogeneity. Heterogeneity has been observed in lung cancer at gene (genetic aberrations) and cellular level through high throughput techniques^61,62^. Careful inspection of PEEPs across LT survivors highlighted specific biological signatures associated with focal adhesion^63^, and extracellular matrix receptors^64^, which helps understand why these patients with PDAC survived longer. Furthermore, it is notable that multiple PDAC responsive pathways^65^ were enriched across several LT survivors and, based on the perturbed gene sets, led to further subgrouping of LT survivors. Understanding these pathways may provide novel insight into the long-term survival mechanism in PDAC. PEEP analysis identified *FCGR3A*, a potential biomarker in PDAC^66^. Two genes, *NOSTRIN* and ADGRG6, were shared by 66% of LTS, and have been reported before to be associated with PDAC survival^52,67^. In independent datset cohort A, *NOSTRIN* gene was found to be shared in atleast two LT survivors.

Drugs bind to their target proteins and may ultimately perturb the transcriptome of a cancer cell^68^. Establishing a causal link between a gene and a disease outcome experimentally remains time-consuming^69^. In our study, analytic functional analysis of individual PEEPs helped to decode homogeneity patterns within LTS. Heterogeneity at the gene level may go hand in hand with homogeneity at the pathway level as different gene perturbations may lead to disruptions in the same molecular pathway. Network-centric approaches resulted in various oncogenes such as *CUL1*, a central component of *SCF*^70^, *EGF*, *FOSL1*^71^, *MMP9*^72^, and *TGFB1*^42^, already known as emerging attractive anticancer targets. Different transcription factors (*GLI2* and *GL3*) were identified, linked to the *KRAS* mechanism of pancreatic tumorigenesis^73^. Identified Immunogenic gene (*CDON*) and epigenetic regulatory gene (*HDAC1*) targets could play significant roles in the future immunotherapeutic strategies in long-term PDAC survivors^58^. *CD8* revealed in our study is in line with recent studies in which *CD8* expression profiling was linked to an immunologic subtype of PDAC with favorable survival^74^. These results, despite the small sample sizes to work with, indicate the possible advantages of employing an integrative analysis pipeline, such as combining knowledge about network-driven disease modules with individual-specific gene perturbation profiling. Unlike DEG-oriented therapeutic target selection for cancers, commonly used to date, we promote the exploitation of analytic frameworks in which multiple network-centric approaches are used for the identification of patient-specific therapeutic targets. This will boost cancer prognosis and treatment in the context of personalized medicine.

## Methods

### Data collection and sequencing

#### Patient selection, ethical statement, and criteria to maximize the definition of STS and LTS

All aspects of the study comply with the Declaration of Helsinki. PDAC patients from Liege University Hospital were recruited based on an opt-out methodology from 2007 to 2014, giving to *N* = 96 pancreas tissue. All participants signed the written informed consent prior to the enrollment. The study was approved by the local institutional ethical board (“Comité d’éthique hospital-faculties universities de Liège) under the reference number B707201627153.

Tissues were obtained from the University of liege Biobank, Belgium. This work is a retrospective study. Between 2007 and 2014, 96 patients were admitted to the CHU Liège for pancreatic cancer. Among the 96 patients, only patients who went a tumour resection were selected to perform RNA sequencing on the tumour tissues. Next, two groups with different statuses of survival were selected: (1) 21 patients who have an overall survival comprised between 3 and 12 months after pancreas cancer diagnosis were selected as the short term survivor group; (2) 15 patients who survived more than 36 months after pancreas cancer diagnosis were selected as the long term survivor group. Patients who died three months after diagnosis or in the period between 12 and 36 months after diagnosis were not included in the study to potentially maximize the molecular differences between long- and short-term survivor groups. We performed RNA extraction from those 36 samples and processed for RNA quality check. The clinical description of patients, treatments and patient outcome is available in supplemental Table S1; Fig. 2A (overall survival curve).

#### RNA extraction, library preparation, sequencing

Tumor areas were determined by a certified pathologist and were manually macro-dissected from the FFPE tissues. RNA was extracted using an All Prep DNA/RNA/miRNA Universal kit (Qiagen, Belgium) according to the manufacturer’s protocol. The RNA quality (*N* = 36) was assessed using a BioAnalyzer (Agilent, Belgium), and the proportion of RNA with a length higher than 200 bases (DV200) was measured. Only 19 out of 36 met a suitable RNA quality, allowing for sequencing. TruSeq® RNA Access Library Prep Kit (Cat. No. RS-301-2001 and RS-301-2002) (Illumina, The Netherlands) was used to prepare libraries, and next-generation sequencing was performed on a NextSeq500 apparatus (Illumina, The Netherlands), in paired-end 2 × 75 bp high output mode.

We performed a series of transcriptome computational analyses to better understand patient heterogeneity between LT and ST survivors. After quality control and adaptor trimming with Trimmomatic^75^, sequence data were mapped to the Genome Reference Consortium GRCh38 assembly using STAR v2.5.2^76^. Read counts for known genes were generated using the function HTSeq-count v0.6.1p^77^ and data were normalized in DESeq2 v1.20.0^78^ as shown in Fig. 1. The study’s analytic workflow is depicted in Fig. 1A–L.

#### Clinical features of patients

Various clinical and pathological parameters of patients (*N* = 19) were included in the analysis. In particular, we collected the following pathological clinical data: age, sex, tumor size, number of lymph nodes evaluated, tumor grade, surgey magins invaded by tumor cells (during or after surgery, a pathologist examines rim of tissue called the surgical margin or margin of resection to be sure it contains no cancer cells), time between surgery and chemotherapy (in days), time between surgery and relapse (in months), disease-free survival (DFS), vascular resection, time in hospital after surgery (in days), re-hospitalization six months after surgery, vascular contact, artery contact, and chemotherapy as shown in Fig. 2A.

### Group-level and Individual-specific analyses

#### Group based DEGs analysis: Differential Gene analysis and functional follow-up

We used DEseq2^78^ for the identification of differentially expressed genes (DEG), with the thresholds log2 fold change ≥ 2 and ≤  − 2, to indicate up-regulation and down-regulation, respectively (Fig. 1B). Significance was assessed at an unadjusted *p*-value < 0.05 in LT versus ST group comparison^79^. We used the ClusterProfiler v3.8.1^80^ package to predict various GO processes enriched in differentially expressed genes (DEGs). To identify the protein domain in DEGs, we used batch CD-Search^81^. For deeper analysis, we downloaded the gene-specific InterPro domains from the Ensembl biomart (https://www.ensembl.org/biomart/martview) database. Further, we performed the enrichment analysis of InterPro domains with DEG in ClusterProfiler v3.8.1 package. Identified DEG was analyzed for detection of prognostic genes, with a log-rank test in a Kaplan–Meier survival model^82^ (Fig. 1A,B). For each gene, patients were classified into two groups, the high-expression group (H), mid-expression group (M) and the low-expression group (L), using the expression median of the gene as a cutoff using the survminer^72^ (v. 0.4.6) R package. Furthermore, to validate identified survival-associated DEGs, we downloaded the three datasets (cohort A: https://www.ncbi.nlm.nih.gov/geo/query/acc.cgi?acc=GSE17891; cohort B: Notta et al. 2016 (https://ega-archive.org/datasets/EGAD00001001956); cohort C: TCGA (https://gdac.broadinstitute.org/). We extracted the processed data of three cohorts with the help of Bioconductor MetaGxPancreas R package (https://bioconductor.org/packages/release/data/experiment/html/MetaGxPancreas.html). All the samples in three cohorts were classified into ST and LT with survival < 12 months and > 36 months and differential gene expression analysis was performed with limma (https://bioconductor.org/packages/release/bioc/html/limma.html) with pvalue < 0.05. Identified DEGs from three cohorts were checked for overlap with significant genes identified from this study.

#### Group-level survival heterogeneity: WGCNA for gene module prediction and assessment of clinical relevance

The minimum sample size to run weighted gene co-expression network analysis (WGCNA) is at least 15. Therefore, WGCNA v1.63^32^ was used on pooled ST and LT PDAC survival patients to generate a transcriptional network from the normalized expression data. The weighted coefficient β was selected based on scale-free topology criteria. The adjacency coefficient α was computed using the power to measure the correlation strength between two genes. The adjacency matrix was created based on α, which was subsequently transformed into a topological overlap matrix (TOM). The distance measure dissTOM = 1 − TOM, served as input to perform average linkage hierarchical clustering (with DynamicTreeCut^83^), giving rise to gene co-expression modules. Gene modules were shown as branches of the resulting pruned tree. It was followed by the calculation of module eigengenes (MEs), defined as the 1st linear principal component of each co-expression module. The hierarchical clustering of MEs was performed to study associations between modules. Approximate non-parametric association tests were used to investigate the association between MEs and PDAC clinical traits. In effect, we used two methods to identify modules related to clinical progression traits. First, within-module gene significance was identified for every module and all available clinical traits. Average gene significance for a module was defined as “module significance”, following recommendations of^84^. Second, rank-based correlation (r) was performed among each ME with the multiple clinic pathological characteristics available in this study (adjusted *p*-value for 0.05 MEs). We used parametric (Pearson correlation coefficient) and non-parametric (Spearman rank) tests for each continuous and categorical data. In order to assess the functional relevance of clinically associated modules, we used ClueGO^85^, a Cytoscape plug-in, to visualize the non-redundant biological terms for genes in a functionally comparative network from multiple clusters. Non-redundancy was assessed via two-sided hypergeometric testing for enrichment/depletion (Bonferroni adjusted *p*-value < 0.05). Cytoscape 5.0^86^ was used for visualizing gene interaction networks (Fig. 1C).

Bailey et al.^26^ reported four subtypes in PDAC i.e. ADEX, Immunogenic, Squamous and Pancreatic Progenitor. We used the SubMap module in GenePattern (https://www.genepattern.org/) to identify the association of studied ST and LT groups to the known PDAC subtypes^26^. Subtypes identified in Puleo et al.^25^ were also used to identify the association with each sample. For this, a centroid-based supervised classification dataset was used and applied to each LT and ST PDAC sample from this study. Next, the correlation coefficients between each sample and the reference subtype centroid were used as a prediction score.

#### Individual-specific survival heterogeneity: quantification of heterogeneity between individual transcriptome profiles, with functional and clinical relevance

We used principles of the PEPPER^24^ method to construct personalized gene expression perturbation profiles for each of *N* = 19 PDAC subjects. PEPPER requires a target class of individuals and a reference class (Fig. 1D). In this study, we took LT PDAC survivors as target group and considered ST survivors as reference (i.e., the most abundant group in real-life). The approach captures the extent to which gene i is perturbed in subject j via a Z-score. This Z-score indicates how many standard deviations the individual’s gene expression is away from the mean value of the reference group. As a threshold, we used |z|= 2. Positive z-scores > 2 would indicate up-regulation, negative z-scores < -2 would indicate down-regulation. Given the small sample sizes to work with in this study, we reshuffled the ST/LT group labels^87^ 500 times and repeated the experiment. The individual LT survivor profiles would be markedly different from average ST survivor profiles under the null hypothesis. Thus LT/ST survivor status would be exchangeable on the basis of individual transcriptome profiles. Functional follow-up analyses included checking for KEGG pathways' enrichment and verifying motif enrichment via ToppGene Suite^88^ (multiple testing adjusted *p*-value < 0.05). Also, patient-specific one-way hierarchical clustering and dendrograms were developed on the basis of the frequency of perturbed genes in identified domains and pathways. Both dendrograms were subsequently compared using the R version 1.12.0 of the dendextend^89^ R package”. For deeper insights, two-way clustering via the superbiclust package in R (RcmdrPlugin.BiclustGUI^90^) version 1.1 was used, enabling the application of the Bimax^90^ algorithm to jointly cluster LT survivors and either one of three levels of biological information, namely gene, pathway and motif levels. A higher level (super) biclustering for each analysis was obtained by constructing a hierarchical tree depicting Jaccard similarity between Bimax clusters.

In the aforementioned PEEPs analyses (PEEP: an individual perturbation expression profile against a reference), no notion of gene-connectivity was used. However, gene connectivity via reference networks can further highlight interesting gene clusters linked to LT survivors. Here, we considered physical interaction data as available from ConsensusPathDB^91^, and obtained 373,101 links between *N* = 19,117 genes. Starting with genes in pathways that already have been implied in PDAC via^68^, and supplementing these genes with searches in the DisGeNet database^34^ (search term = “Pancreatic Diseases”), resulted in 53 seed genes (Fig. 1I). We then used DADA’s module detection algorithm^6^ to augment the initial list of 53 seed genes and identify PDAC disease modules. The top 1 percent highest-ranked genes were considered to form a disease module. Significantly perturbed genes (in LT survivor PEEPs) were mapped on the identified disease module. This allowed putting LT survival individual specific genes in the context of gene connectivity and gene neighborhoods. All DADA top 1 percent genes were checked for retrieval in previous analyses (Fig. 1J,L). As an alternative approach to exploit gene interaction network structure, we adapted NetICS^92^, an approach initially intended to prioritize cancer genes on a directed functional interaction network. It uses an individual-specific list of genes via bidirectional network diffusion of two layers of information (Fig. 1E). As the first layer, we took the individual-specific significant genes as highlighted in the LT PDAC survival PEEPs analyses before (instead of mutant genes per sample in the original NetICS implementation). As second layer we took groups specific DEGs. Individual-specific gene ranks (for LT survivors) were aggregated via NetICS methodology into an overall ranked list of genes, with restart probability of 0.4. The top 1% percent ranked genes were retained. Similar to follow-up of DADA top-ranked genes, we checked for the frequency of NetICS derived top-ranked genes that were also retrieved in former analyses (Fig. 1F–H).

## Conclusion

In this study, we performed a series of transcriptome computational analyses to better understand PDAC survival heterogeneity. To our knowledge, we demonstrated and applied for the first time in PDAC samples an integrative analytic workflow, combining clinical and omics data, and individual-level and group-level transcriptome profiling. In addition, we showed the utility of network-based approaches, disease modules and multi-scale functional analyses (gene, protein domain, pathway), that led to the identification of known oncogenes and genes with promising therapeutic potential, as well as genes that highlighted gene-level heterogeneity among long-term PDAC survivors. From both the group and individual level analysis, we found various gene targets and their role in immune specific pathways in PDAC survival mechanism. Hence, all the analysis confirms the role of immune specific pathways as potential therapeutic targets for PDAC survival.

## Softwares used

All analyses have been conducted according to software packages discussed in the method section. We have utilized the following software packages in our present study DESeq2^78^ (differential analysis at group level), WGCNA^32^ (module detection in gene expression data), PEPPER^24^ (differential analysis at individual level), ClusterProfiler^80^ v3.8 (functional analysis of differential genes), survminer^93^ (v. 0.4.6) R package (development of survival plot), Cytoscape^86^ 5.086 and ToppGene^88^ Suite90 (functional annotation of genes), DisGeNet^34^ (retrieval of disease associated gene list); biclustGUI^90^ (biclustering of genes), DADA^7^ (development of disease associated network module). Next, used the matlab based software NetICS for the integration of group- and individual-level integration. GSVA v1.40.187. For visualization of heatmap, used pheatmap^94^ v1.0.1288.

## Supplementary Information

Supplementary Information.

## Data Availability

Data deposited in GEO with accession number GSE150043.
