# Supplementary material for: Deeper insights into long-term survival heterogeneity of pancreatic ductal adenocarcinoma (PDAC) patients using integrative individual- and group-level transcriptome network analyses
Source: Sci Rep. 2022 Jun 30;12:11027. doi: 10.1038/s41598-022-14592-1 (PMC9247075; doi:10.1038/s41598-022-14592-1)

Supplementary Methods

Deeper insights into long-term survival heterogeneity of Pancreatic Ductal Adenocarcinoma (PDAC) patients using integrative individual- and group-level transcriptome network analyses

Archana Bhardwaj[a], Claire Josse[b,c], Daniel Van Daele[d], Christophe Poulet[b,g], Marcela Chavez[e], Ingrid Struman[f], Kristel Van Steen[a,]

[a]GIGA-R Centre, BIO3 – Medical Genomics, University of Liège, Liège, Belgium

[b]Laboratory of Human Genetics, GIGA Research, University Hospital (CHU), Liège, Belgium

[c]Medical Oncology Department, CHU Liège, Liège, Belgium

[d]Department of Gastro-enterology, University Hospital (CHU), Liège, Belgium

[e]Department of Medicine, Division of Hematology, University Hospital (CHU), Liège, Belgium

[f]GIGA-R Centre, Laboratory of Molecular Angiogenesis, University of Liège, Liège, Belgium

[g]Laboratory of Rheumatology, GIGA-R, University Hospital (CHULiege), Liège, Belgium

Contact Detail: Corresponding author

University of Liège

GIGA - Quartier Hôpital

Avenue de l’Hôpital, 11

4000 Liège, Belgium

Table S1. Complete list of all sample (patients) and their clinical features as considered in the present study.

**Patient ID**	**Group***	**Age at the surgery (years)**	**Sex[a]**	**Tumor size (cm)[b] (measured after surgery)**	**T**	**N**	**Number of nodes analyzed**
S1	ST	74	F	3	4	1	25
S2	ST	71	M	3.5	3	0	12
S3	ST	72	F	3.2	3	0	7
S4	ST	73	M	4	3	1	7
S5	ST	54	F	3.2	4	1	12
S6	ST	80	M	4	3	1	8
S7	ST	80	M	4	3	0	16
S8	ST	63	F	3	4	1	14
S9	ST	66	M	5	3	1B	16
S10	ST	66	M	5	4	1	4
L1	LT	78	F	2.5	3	1	13
L2	LT	58	M	3.2	3	0	33
L3	LT	71	F	2	3	0	10
L4	LT	83	M	4	3	0	14
L5	LT	67	M	2.2	3	1	25
L6	LT	62	M	5	3	1	7
L7	LT	58	M	2	1	1	8
L8	LT	77	M	4.5	4	1b	4
L9	LT	55	M	0.6	1	0	0

Complete list of all sample (patients) and their clinical features analyzed in the present study (Table S1 continue)

Patient ID	ratio node (invaded/analyzed)	ratio node % (invaded/analysed)	Sugey Magins invaded by tumor cells**	Time between surgery and chemotherapy (in days)	Time between surgery and relapse (months) (DFS)[c]	Time between relapse and death (months)	Death	Vascular resection
S1	2/25	0.08	1	82	NA	NA	1	1
S2	0/12	0.00	0	NA	5.00	1.31	1	1
S3	0/7	0.00	0	71	NA	NA	1	0
S4	5/7	0.71	0	54	5.42	2.89	1	0
S5	7/12	0.58	0	63	2.30	6.11	1	0
S6	2/8	0.25	1	74	4.50	4.11	1	0
S7	0/16	0.00	0	NA	6.87	2.79	1	0
S8	4/14	0.28	1	43	4.87	4.80	1	1
S9	10/16	0.62	0	53	7.10	2.66	1	0
S10	0/4	0.00	0	41	9.37	0.85	1	0
L1	4/13	0.31	0	55	25.25	NA	1	0
L2	0/33	0.00	2	NA	17.16	23.54	1	1
L3	0/10	0.00	1	NA	NA	NA	1	0
L4	0/14	0.00	0	NA	NA	NA	1	0
L5	2/25	0.08	0	16	NA	NA	0	0
L6	1/7	0.14	0	71	37.71	22.81	1	0
L7	3/8	0.37	0	NA	NA	NA	0	0
L8	3/4	0.75	0	66	NA	NA	0	0
L9	0	NA	0	NA	NA	NA	0	0

Complete list of all sample (patients) and their clinical features analyzed in the present study (Table S1 continue)

Patient ID	Time in hospital after surgery (in days)	Re-hospitalisation 6 months after surgery**	Vascular contact (if tumor contact a vein (often portal vein)**	artery contact (if tumor contact an artery) **	Tumor size by imagery (mm)[e]	Time between imagery and surgery (days)	Adjuvant chemotherapy (CTH)	CTH > 3 months	Number of CTH cures	Dose CTH received in % of the maximal theoretical value
S1	42	0	1	0	42	7	1	0	1	100
S2	14	0	0	0	76	121	0	NA	NA	NA
S3	16	0	NA	NA	NA	NA	1	0	NA	NA
S4	22	3	1	0	22	6	1	0	1	100
S5	16	0	1	0	29	6	1	0	4	100
S6	24	0	1	1	34	5	1	0	6	80
S7	21	1	1	1	27	3	0	NA	NA	NA
S8	18	0	1	0	NA	31	1	0	7	100
S9	24	0	1	1	19	21	1	0	3	100
S10	17	0	1	0	NA	92	1	0	2	NA
L1	22	0	1	0	15	9	1	0	4	100
L2	14	1	NA	NA	NA	NA	1	1	NA	NA
L3	11	0	1	0	28	22	0	NA	NA	NA
L4	22	0	1	0	NA	27	0	NA	NA	NA
L5	17	0	1	0	28	33	1	0	NA	NA
L6	17	0	0	0	NA	63	1	0	4	75
L7	13	0	1	1	21	28	NA	NA	NA	NA
L8	28	0	1	0	41	28	1	0	18	100
L9	21	0	1	0	21	28	0	NA	NA	NA

[*] ST=short-term PDAC survivor (ST; death >=3 months et <12 months) ; LT= long-term PDAC survivor (LT; alive or death >=36 months) ; [a]F= Female ; M=Male ; [b]cm = centimeters ; [c]DFS=Disease free survival ; [d]OS= Overall survival ; [e]mm= millimeters ; NA =Not available; **(1=yes;0=no)

Table S2 : List of differential expressed genes classified based on the Up (log2foldchange >2) and Down (<-2 log2Foldchange) from the DESeq2 analysis.

Gene	p-value	Group
ENSG00000169605	1.25E-05	up
ENSG00000132164	0.02784500215	up
ENSG00000145384	0.01343692457	up
ENSG00000224721	0.01104201319	up
ENSG00000200726	0.0388922003	up
ENSG00000160868	0.0009047386938	up
ENSG00000214300	0.009802336193	up
ENSG00000196834	0.00801747567	up
ENSG00000116745	0.01836934549	up
ENSG00000197454	0.04420540202	up
ENSG00000200503	0.002757277905	up
ENSG00000133124	0.01292947042	up
ENSG00000211656	0.00200495285	up
ENSG00000215148	0.03663189627	up
ENSG00000070019	0.01437320677	up
ENSG00000260494	0.0265987158	up
ENSG00000131668	0.02123002913	up
ENSG00000183793	0.001038081435	up
ENSG00000230593	0.02905323412	up
ENSG00000036473	0.002321798267	up
ENSG00000258646	0.04890024946	up
ENSG00000271375	0.001299692795	up
ENSG00000177414	0.01052210495	up
ENSG00000109101	0.01012122454	up
ENSG00000200593	0.003028887042	up
ENSG00000179066	0.02160429766	up
ENSG00000057149	0.01364838994	up

ENSG00000165799	0.01156519372	up
ENSG00000129991	0.02853273069	up
ENSG00000239819	0.003334718473	up
ENSG00000134115	0.01101310595	up
ENSG00000249948	1.08E-05	up
ENSG00000224114	0.003657770062	up
ENSG00000118492	0.01614629011	up
ENSG00000144285	0.02617901927	up
ENSG00000189377	0.000356662211	up
ENSG00000232216	0.0001781618629	up
ENSG00000169427	0.04075570246	up
ENSG00000184611	0.007085814927	up
ENSG00000125931	0.02440175225	up
ENSG00000077279	0.01838229232	up
ENSG00000205918	0.02192005506	up
ENSG00000242371	0.01383667365	up
ENSG00000104371	0.02679406105	up
ENSG00000006128	0.008441504313	up
ENSG00000181072	0.02783912381	up
ENSG00000161055	0.03608471756	up
ENSG00000162078	0.001022093342	up
ENSG00000237541	0.005699299624	up
ENSG00000124253	1.20E-05	up
ENSG00000240671	0.04435722148	up
ENSG00000198944	0.007578632334	up
ENSG00000185306	0.01633248596	up
ENSG00000229619	0.00651168161	up
ENSG00000166391	0.04715297843	up
ENSG00000118298	0.04273618901	up
ENSG00000166796	0.04606812179	up
ENSG00000184923	0.03516737597	up

ENSG00000205810	0.03340524998	up
ENSG00000239975	0.003522704716	up
ENSG00000137878	0.007443924953	up
ENSG00000184515	0.000826584352	up
ENSG00000134193	7.85E-05	up
ENSG00000257818	0.02861876576	up
ENSG00000163586	0.04796247418	up
ENSG00000229089	0.03884345258	up
ENSG00000090382	0.001850647464	up
ENSG00000205592	0.02184456286	up
ENSG00000164049	0.04270016089	up
ENSG00000122585	0.04869389187	up
ENSG00000277856	0.00596724403	up
ENSG00000223394	0.02797442262	up
ENSG00000239470	0.01038528008	up
ENSG00000197888	0.03550313013	up
ENSG00000129204	0.01235297014	up
ENSG00000096088	0.04129630103	up
ENSG00000239855	0.01452793315	up
ENSG00000105388	0.02567189713	up
ENSG00000121716	0.009417325671	up
ENSG00000100433	0.03691825387	up
ENSG00000144460	0.03922103515	up
ENSG00000198535	0.003609719134	up
ENSG00000066405	0.008394679444	up
ENSG00000160182	0.008340724706	up
ENSG00000116299	0.0002793367633	up
ENSG00000197520	0.001666334896	up
ENSG00000130957	0.03973487998	up
ENSG00000127324	0.0003403716792	up
ENSG00000112280	0.01138592497	up

ENSG00000211632	0.001502715062	up
ENSG00000265574	0.0458104974	up
ENSG00000100473	0.001199129253	up
ENSG00000232801	0.02770958291	up
ENSG00000204616	0.004346648516	up
ENSG00000217094	0.0006928336582	up
ENSG00000128965	0.006415828225	up
ENSG00000213058	0.02703104013	up
ENSG00000079112	0.02225681354	up
ENSG00000183607	0.03732252958	down
ENSG00000204805	0.02227945156	down
ENSG00000206688	4.58E-05	down
ENSG00000181378	0.003383729915	down
ENSG00000225523	0.0182739803	down
ENSG00000096006	0.018037425	down
ENSG00000160181	0.01072035161	down
ENSG00000197753	0.0472951303	down
ENSG00000118160	0.003003730372	down
ENSG00000280071	0.001495790403	down
ENSG00000113249	0.001195801014	down
ENSG00000182308	0.001526545351	down
ENSG00000138271	3.32E-06	down
ENSG00000197233	0.04751573696	down
ENSG00000186847	0.004650394483	down
ENSG00000213857	0.02781457015	down
ENSG00000147588	0.03275018669	down
ENSG00000279058	0.004692187948	down
ENSG00000075461	0.001811246878	down
ENSG00000174715	0.01108439004	down
ENSG00000213937	0.01167166661	down
ENSG00000126500	0.003493268033	down

ENSG00000225871	0.01318706782	down
ENSG00000243444	0.0407701535	down
ENSG00000137745	0.002181422633	down
ENSG00000204020	0.00224010047	down
ENSG00000147697	0.006361321955	down
ENSG00000181143	0.0174888778	down
ENSG00000167646	0.004341768922	down
ENSG00000186684	0.001476176834	down
ENSG00000214249	0.004599226821	down
ENSG00000227827	0.006010740328	down
ENSG00000269318	0.01264918613	down
ENSG00000167755	0.01854975695	down
ENSG00000265190	0.02361970536	down
ENSG00000138675	0.03322594065	down
ENSG00000187475	0.02852427599	down
ENSG00000174792	0.04162766997	down
ENSG00000203886	0.03697995961	down
ENSG00000166763	0.003473938699	down
ENSG00000100146	0.003839332555	down
ENSG00000181773	0.00652614876	down
ENSG00000207931	0.009427296402	down
ENSG00000175315	0.000574875517	down
ENSG00000211581	0.002127977642	down
ENSG00000235670	0.02495624261	down
ENSG00000128610	0.04765813198	down
ENSG00000172478	0.001035586048	down
ENSG00000275591	0.01256054399	down
ENSG00000196844	0.009358604567	down
ENSG00000104055	0.009859265616	down
ENSG00000168843	0.02540928053	down
ENSG00000221227	0.0006549068875	down

ENSG00000257017	0.001225570323	down
ENSG00000238084	0.01361513331	down
ENSG00000254681	9.67E-06	down
ENSG00000273777	0.0233453197	down
ENSG00000112041	0.04811006031	down
ENSG00000197353	0.04063448049	down
ENSG00000257506	0.01801070877	down
ENSG00000253161	0.01216692224	down
ENSG00000004939	0.01589005788	down
ENSG00000164393	0.01779954919	down
ENSG00000282639	0.02009930903	down
ENSG00000196754	1.75E-05	down
ENSG00000181355	0.002179754485	down
ENSG00000237433	0.01932650442	down
ENSG00000205420	0.001370492174	down
ENSG00000112761	0.003138818226	down
ENSG00000213030	0.002112641428	down
ENSG00000110244	0.02077338998	down
ENSG00000189052	0.00893681441	down
ENSG00000019186	0.006555056067	down
ENSG00000250696	0.005507793313	down
ENSG00000230937	0.00873824419	down

Table S3. Significant enriched gene ontology identified from differential expressed gene. Cluster indicates up and down regulated genes categories based on their log2fold change value.

Cluster	ONTOLOGY	ID	Description	p.adjust
down	MF	GO:0005546	phosphatidylinositol-4,5-bisphosphate binding	0.0286154331481165
down	MF	GO:0043178	alcohol binding	0.0286154331481165
down	MF	GO:1902936	phosphatidylinositol bisphosphate binding	0.0353540918789734
down	MF	GO:0005543	phospholipid binding	0.0359328722103267
up	BP	GO:0007586	digestion	1,08E+09
up	BP	GO:0002377	immunoglobulin production	0.0011483962664695
up	BP	GO:0022600	digestive system process	0.00188657499270426
up	BP	GO:0002440	production of molecular mediator of immune response	0.00439178106593195
up	BP	GO:0001894	tissue homeostasis	0.0257049503703018
up	CC	GO:0019814	immunoglobulin complex	5,36E+07

Table S4. List of prognostic survival (ENSMBL ID) associated genes

ENSG00000280071	ENSG00000211581
ENSG00000221227	ENSG00000187475
ENSG00000181355	ENSG00000213937
ENSG00000250696	ENSG00000205420
ENSG00000175315	ENSG00000166763
ENSG00000112761	ENSG00000004939
ENSG00000100146	ENSG00000124253
ENSG00000225871	ENSG00000198944
ENSG00000269318	ENSG00000200503
ENSG00000203886	ENSG00000206688
ENSG00000126500	ENSG00000109101
ENSG00000182308	ENSG00000169605
ENSG00000147588	ENSG00000257818
ENSG00000167646	ENSG00000223394
ENSG00000213030	ENSG00000239470
ENSG00000113249	ENSG00000184611
ENSG00000167755	ENSG00000169427
ENSG00000104055	ENSG00000006128
ENSG00000275591	ENSG00000118298
ENSG00000174715	ENSG00000121716
ENSG00000265190	ENSG00000116745
ENSG00000204020	ENSG00000166796
ENSG00000172478	ENSG00000184515
ENSG00000196844	ENSG00000205592
ENSG00000253161	
ENSG00000230937	
ENSG00000227827	
ENSG00000254681	
ENSG00000213857	

Table S5. Pathways enriched in LT survivors based on the Individual specific PEEPs annotated using ToppGene Suite[17] (section 2.2). For all identified significant pathways, corresponding Bonferroni corrected p-values are shown. Identified pathways are grouped into various categories based on functional annotation. highlighted based on their involvement either in three LT survivors: two LT survivors or one LT survivor.

Pathway Name	LT2	LT7	LT9	Category
Apoptosis			1.20E-02	Cell regulation
Caspase cascade in apoptosis			7.85E-03	
Cell Cycle			8.98E-03	
Cell Cycle. Mitotic			5.48E-03	
Cell junction organization			9.15E-03	
Cell surface interactions at the vascular wall			4.99E-06	
Cell-Cell communication			3.04E-06	
ECM-receptor interaction	3.53E-02	3.01E-02	3.22E-05	
Endocytosis			3.05E-02	
Mitotic Anaphase			1.06E-02	
Mitotic Metaphase and Anaphase			1.19E-02	
Mitotic Prometaphase			1.10E-03	
Programmed Cell Death			6.95E-03	
Regulation of actin dynamics for phagocytic cup formation			2.88E-03	
Semaphorin interactions			9.86E-05	
Axon guidance	2.25E-07	3.01E-02	3.92E-19	Developmental Biology
EPH-ephrin mediated repulsion of cells	3.52E-02			
L1CAM interactions			2.37E-02	
Osteoclast differentiation			1.91E-05	
Other semaphorin interactions			5.48E-03	
Scavenging by Class A Receptors		3.24E-03		
AGE-RAGE signaling pathway in diabetic complications			1.53E-06	Disease
Amoebiasis			1.42E-04	
Amyotrophic lateral sclerosis (ALS			4.32E-02	
Angiogenesis			1.81E-04	

Arrhythmogenic right ventricular cardiomyopathy (ARVC			1.66E-02	
Chagas disease (American trypanosomiasis			1.96E-04	
HTLV-I infection			1.34E-04	
Hypertrophic cardiomyopathy (HCM			2.01E-04	
Inflammatory bowel disease (IBD			9.65E-04	
Influenza A			2.68E-02	
Legionellosis			1.43E-03	
Leishmaniasis			3.34E-04	
Malaria			1.29E-03	
Measles			3.05E-02	
Pathogenic Escherichia coli infection			2.10E-04	
PDGFR-beta signaling pathway			2.08E-02	
Pertussis			1.46E-07	
Primary immunodeficiency			2.30E-02	
Prolonged ERK activation events			1.99E-02	
RHO GTPases Activate Formins			2.01E-05	
Salmonella infection			1.50E-03	
SHC1 events in EGFR signaling			2.30E-02	
SRP-dependent cotranslational protein targeting to membrane			7.42E-03	
Toll-Like Receptors Cascades			1.75E-07	
TRIF-mediated TLR3/TLR4 signaling			4.72E-03	
Tuberculosis			4.00E-05	
Assembly of collagen fibrils and other multimeric structures		3.70E-04	5.76E-12	
Collagen biosynthesis and modifying enzymes		3.01E-04	6.80E-06	
Collagen chain trimerization		3.01E-04	3.74E-07	
Collagen degradation			1.49E-02	
Collagen formation		1.41E-03	4.36E-11	Extracellular matrix organization
Degradation of the extracellular matrix			3.53E-04	
ECM proteoglycans			1.03E-04	
Elastic fibre formation	8.69E-03			

Ensemble of genes encoding core extracellular matrix including ECM glycoproteins. collagens and proteoglycans	3.18E-03	3.01E-02	1.47E-03	
Ensemble of genes encoding extracellular matrix and extracellular matrix-associated proteins	3.23E-03		4.51E-05	
Extracellular matrix organization	2.25E-07	1.40E-09	2.38E-19	
Genes encoding collagen proteins		2.66E-04	9.34E-08	
Genes encoding structural components of basement membranes	5.52E-03		3.51E-04	
Integrin cell surface interactions		1.16E-02	3.63E-06	
Laminin interactions			1.91E-05	
Molecules associated with elastic fibres	3.57E-02			
Non-integrin membrane-ECM interactions			8.72E-05	
Activated TLR4 signalling			1.83E-04	Immune
Adaptive Immune System			2.25E-04	
Adhesion and Diapedesis of Granulocytes			2.41E-03	
Adhesion and Diapedesis of Lymphocytes			2.93E-03	
amb2 Integrin signaling			4.70E-03	
Bacterial invasion of epithelial cells			3.46E-02	
Cells and Molecules involved in local acute inflammatory response			1.68E-02	
Chemokine signaling pathway			2.94E-06	
Classical Complement Pathway			2.41E-03	
Complement and coagulation cascades			8.27E-05	
Complement Pathway			4.69E-02	
CTL mediated immune response against target cells			5.04E-03	
CXCR4-mediated signaling events			1.67E-04	
Cytokine Signaling in Immune system			1.04E-16	
Cytokine-cytokine receptor interaction			1.43E-03	
DAP12 interactions			3.75E-06	
DAP12 signaling			2.82E-05	
Dilated cardiomyopathy			3.04E-06	
Direct p53 effectors			1.06E-03	
Diseases of signal transduction			1.94E-02	
Fc gamma R-mediated phagocytosis			1.12E-03	

FCERI mediated MAPK activation			2.13E-03	
Fcgamma receptor (FCGR dependent phagocytosis			2.32E-03	
Hematopoietic cell lineage			1.06E-02	
IL-2 Receptor Beta Chain in T cell Activation			3.05E-02	
IL12 signaling mediated by STAT4			9.13E-04	
IL12-mediated signaling events			3.13E-04	
IL4-mediated signaling events			9.64E-03	
Inactivation of Gsk3 by AKT causes accumulation of b-catenin in Alveolar Macrophages			2.61E-02	
Inflammation mediated by chemokine and cytokine signaling pathway			8.51E-04	
Innate Immune System			1.11E-14	
Interleukin-1 signaling			8.72E-05	
Interleukin-10 signaling			6.00E-03	
Interleukin-2 signaling			9.95E-03	
Interleukin-3. 5 and GM-CSF signaling			2.13E-03	
Interleukin-4 and 13 signaling			3.30E-05	
Leukocyte transendothelial migration			2.13E-07	
Monocyte and its Surface Molecules			2.93E-03	
MyD88 dependent cascade initiated on endosome			5.48E-03	
MyD88-independent TLR3/TLR4 cascade			4.72E-03	
MyD88:Mal cascade initiated on plasma membrane			2.32E-03	
Natural killer cell mediated cytotoxicity			3.44E-03	
NCAM signaling for neurite out-growth			1.48E-03	
Neutrophil degranulation			1.26E-05	
NOD-like receptor signaling pathway			7.77E-03	
PLK1 signaling events			1.43E-03	
Regulation of actin cytoskeleton			4.33E-10	
Signaling by EGFR			1.41E-04	
Syndecan-4-mediated signaling events			6.29E-06	
T cell activation			3.74E-05	
T cell receptor signaling pathway			2.84E-03	
T Cell Signal Transduction			9.42E-03	

T Cytotoxic Cell Surface Molecules			2.41E-03	
T Helper Cell Surface Molecules			1.64E-04	
TCR signaling in naive CD4+ T cells			4.82E-05	
Th1 and Th2 cell differentiation			1.38E-03	
Th17 cell differentiation			9.43E-06	
TNF signaling pathway			2.13E-03	
Toll Like Receptor 2 (TLR2 Cascade			2.32E-03	
Toll Like Receptor 3 (TLR3 Cascade			4.72E-03	
Toll Like Receptor 4 (TLR4 Cascade			8.90E-06	
Toll Like Receptor 7/8 (TLR7/8 Cascade			5.48E-03	
Toll Like Receptor 9 (TLR9 Cascade			1.06E-02	
Toll Like Receptor TLR1:TLR2 Cascade			2.32E-03	
Toll Like Receptor TLR6:TLR2 Cascade			2.32E-03	
Toxoplasmosis			3.52E-05	
TRAF6 mediated induction of NFkB and MAP kinases upon TLR7/8 or 9 activation			1.27E-02	
Aurora B signaling			4.13E-02	other
Binding and Uptake of Ligands by Scavenger Receptors			2.63E-02	
Chylomicron-mediated lipid transport		2.93E-02		
GPVI-mediated activation cascade			1.10E-03	
Hemostasis			2.62E-16	
ARMS-mediated activation			3.29E-02	Signal transduction
Cell adhesion molecules (CAMs			2.42E-04	
Downstream signal transduction			2.26E-04	
EGF receptor signaling pathway			1.02E-02	
EPH-Ephrin signaling	3.53E-02		6.89E-04	
Fc epsilon receptor (FCERI signaling			2.20E-04	
Focal adhesion	3.57E-02	3.24E+03	1.95E-11	
Frs2-mediated activation			1.67E-02	
Gastrin-CREB signaling pathway via PKC and MAPK			4.35E-03	
GRB2 events in EGFR signaling			2.30E-02	
Hippo signaling pathway			1.98E-02	

HIV-I Nef: negative effector of Fas and TNF			1.25E-02	
Insulin receptor signalling cascade			2.23E-02	
integrin signaling			1.10E-03	
Integrin signaling pathway	3.57E-02	1.16E-02	6.85E-04	
Interleukin signaling pathway			4.72E-03	
MAPK family signaling cascades			1.11E-03	
MAPK1/MAPK3 signaling			8.25E-03	
MET activates PTK2 signaling			9.28E-03	
MET promotes cell motility			1.85E-02	
mTOR signaling pathway			2.41E-02	
NGF signaling via TRKA from the plasma membrane			2.16E-03	
Pathways in cancer	8.93E-01	1.95E-02	1.40E-13	
Phagosome			1.45E-05	
Phospholipase D signaling pathway			7.42E-03	
PI3K-Akt signaling pathway	9.26E-02	3.76E+03	4.13E-07	
Platelet activation			2.21E-04	
Platelet activation. signaling and aggregation			8.93E-06	
Proteoglycans in cancer	9.26E-02		7.31E-08	
RAF/MAP kinase cascade			2.30E-02	
Rap1 signaling pathway			7.70E-04	
Ras Pathway			2.68E-02	
Resolution of Sister Chromatid Cohesion			9.15E-03	
RET signaling			4.35E-03	
RHO GTPase Effectors			2.43E-06	
Separation of Sister Chromatids			1.97E-02	
Shigellosis			4.19E-05	
Signaling by Interleukins			3.53E-15	
Signaling by Leptin			9.73E-03	
Signaling by MET			1.30E-04	
Signaling by PDGF			2.80E-06	
Signaling by Rho GTPases			1.75E-07	

Signaling by SCF-KIT			5.53E-05	
Signaling by VEGF			1.56E-03	
Signaling events mediated by focal adhesion kinase			1.10E-03	
Signaling by NGF			1.26E-05	
Signaling to ERKs			2.37E-02	
Signaling to p38 via RIT and RIN			1.51E-02	
Small cell lung cancer			2.42E-04	
SOS-mediated signaling			2.30E-02	
Staphylococcus aureus infection			5.76E-06	
Syndecan-2-mediated signaling events			3.50E-02	
Tight junction			1.34E-04	
Validated transcriptional targets of AP1 family members Fra1 and Fra2			9.02E-03	
VEGFA-VEGFR2 Pathway			7.65E-03	
VEGFR2 mediated cell proliferation			3.31E-02	

Table S6. Protein domains enriched in LT survivors based on the Individual specific PEEPs annotated using ToppGene Suite[17] (section 2.2). For all identified significant protein domains, corresponding Bonferroni corrected p-values are shown. Identified protein domains are ordered based on similar annotation profile as given below. (Only 3 LT survivors showed enriched IPR domains).

**Domain ID**	**LT2**	**LT7**	**LT9**	**Description**
PS00010	0.000368			ASX_HYDROXYL
PF12662	0.000247			cEGF
IPR026823	0.000247			cEGF
SM00038		2.5E-05		COLFI
PF01410		2.5E-05		COLFI
PF01391		0.001874	4.19E-06	Collagen
IPR008160		0.001874	4.19E-06	Collagen
IPR011029			0.00017	DEATH-like_dom
SM00181	2.1E-06	7.05E-05	1.5E-06	EGF
PF00008			9.42E-06	EGF
PS00022	8.89E-05	5.77E-05	4.19E-06	EGF
PS01186	9.45E-06	4.21E-06	7.02E-06	EGF
PS50026	0.000011		0.000209	EGF
PF07645	5.51E-05	0.000249		EGF
SM00179	2.47E-05	0.001874		EGF
PS01187	5.01E-05			EGF
IPR018097	4.01E-05			EGF_Ca-bd_CS
IPR001881	2.9E-05	0.002076		EGF-like_Ca-bd_dom
IPR013032	2.16E-06	1.02E-05	4.79E-08	EGF-like_CS
IPR000742	9.45E-06	4.12E-05	7.07E-04	EGF-like_dom
IPR000152	0.000121			EGF-type_Asp/Asn_hydroxyl_site
PD002078		2.5E-05		Fib_collagen_C
IPR000885		2.5E-05		Fib_collagen_C
SM00060			0.000799	FN3
PS50853			0.004262	FN3
IPR003961			0.000334	FN3_dom

IPR009030	9.37E-05	0.005339	1.59E-05	Growth_fac_rcpt_
PS00856	0.02688			GUANYLATE_KINASE_1
PS50052	0.02688			GUANYLATE_KINASE_2
IPR013783			4.15E-05	Ig-like_fold
IPR032695			0.0002	Integrin_dom
IPR011009	0.04356			Kinase-like_dom
IPR001791			8.36E-05	Laminin_G
2.60.40.10			4.19E-06	NA*
1.10.533.10			0.000225	NA
PS51461		2.5E-05		NC1_FIB
PF07714	0.04519			Pkinase_Tyr
IPR000719	0.00585			Prot_kinase_dom
PS00107	0.001612		0.00355	PROTEIN_KINASE_ATP
IPR017441	0.01304			Protein_kinase_ATP_BS
PS50011	0.006695			PROTEIN_KINASE_DOM
PS00109	0.01171			PROTEIN_KINASE_TYR
PS50001			0.005654	SH2
IPR008266	0.01171			Tyr_kinase_AS
IPR020635	0.004333			Tyr_kinase_cat_dom
SM00219	0.004333			TyrKc
PS01208	0.04908			VWFC_1

*NA = Not available

Table S7. Seed genes for the identification of a PDAC disease module with DADA[13]. Genes are ordered following their genetic location; genes annotation shown as well.

**Gene**	**Chromosome**	**Description**
HDAC1	1	histone deacetylase 1
GLI2	2	GLI family zinc finger 2
TGFA	2	transforming growth factor alpha
IHH	2	Indian hedgehog signaling molecule
AREG	4	amphiregulin
HHIP	4	hedgehog interacting protein
EGF	4	epidermal growth factor
MAML3	4	mastermind like transcriptional coactivator 3
APC	5	APC regulator of WNT signaling pathway
HDAC2	6	histone deacetylase 2
CCND3	6	cyclin D3
EGFR	7	epidermal growth factor receptor
RAC1	7	Rac family small GTPase 1
SHH	7	sonic hedgehog signaling molecule
MYC	8	MYC proto-oncogene@ bHLH transcription factor
DKK4	8	dickkopf WNT signaling pathway inhibitor 4
DKK1	10	dickkopf WNT signaling pathway inhibitor 1
FRAT1	10	FRAT regulator of WNT signaling pathway 1
CCND1	11	cyclin D1
ATM	11	ATM serine/threonine kinase
MMP7	11	matrix metallopeptidase 7
FOSL1	11	FOS like 1@ AP-1 transcription factor subunit
INHBE	12	inhibin subunit beta E
GLI1	12	GLI family zinc finger 1
WIF1	12	WNT inhibitory factor 1
IFNG	12	interferon gamma
ZIC2	13	Zic family member 2

JAG2	14	jagged canonical Notch ligand 2
SMAD3	15	SMAD family member 3
DLL4	15	delta like canonical Notch ligand 4
MAPK3	16	mitogen-activated protein kinase 3
SMAD4	18	SMAD family member 4
SMAD2	18	SMAD family member 2
SMAD7	18	SMAD family member 7
TGFB1	19	transforming growth factor beta 1
BMP2	20	bone morphogenetic protein 2

Table S8. Overlap between individual perturbation expression profile (PEEP) for LT PDAC survivors with top 1% gene list from NetICS and DADA. NetICS= Network-based Integration of Multi-omics Data[24]; DADA= Degree-Aware Algorithms for Network Based Disease Gene Prioritization[13].

**Identified in NetICS**		
**Patient ID**	**Nr* of PEEP genes in top 1% NetICD list**	**Top 5 genes**
L1	1	TNNI3
L2	143	TNNI3.IRS4.ACTN2.MAPK14.PLCG1.
L3	5	FOSL1.MYL2.FCGR3A.USF2.NOSTRIN
L4	38	ACTN2.PRKCA.ITGA8.AP2A1.NRAS
L5	20	TJP2.ITGA10.SDC4.TYROBP.SPI1
L6	26	NPY.MPDZ.CNTN6.ITGB4.TNNC1
L7	119	TNNI3.HLA-DQA1.APOA4.IRS4.NPY.
L8	18	GNAI3. ITGA10. KLRC3. SPI1.TNNC1
L9	336	ACTB. ACTG1.NFKB1.ITGB1.CTNNB1
**Identified in DADA**		
**Patient ID**	**Nr of PEEP genes in top 1% DADA list**	**Top 5 genes**
L2	8	GLI2.RAC1.CUL1.ESR1.FBLN1.UBC.WNT11
L3	1	FOSL1
L4	3	TGFA.EGF.CDK2
L5	2	UBC.CDON
L7	9	ESR1.GLI2.WNT11.GLI3.HDAC1.CDK2.UBC.CDON
L9	12	GLI2.GLI3.UBC.RAC1.ACTB.AREG.DKK1.GRB2.MMP9.TGFB1.EGF.FOSL1

*Nr=Number

Table S9. Overlap between top 1% genes from NetICS /DADA and clinical modules, as defined in section 2.1 and 2.2. WGCNA = Weighted correlation network analysis[10]; NetICS= Network-based Integration of Multi-omics Data[24]; DADA= Degree-Aware Algorithms for Disease Gene Prioritization[13] .

Category	Module No.	Gene Symbol	Chromosome	Gene Description
**Common among NetICS AND WGCNA**	M30	CDH1	16	cadherin 1
	M30	CREB3L3	19	cAMP responsive element binding protein 3 like 3
	M30	NGFR	17	nerve growth factor receptor
	M30	CDON	11	cell adhesion associated oncogene regulated
	M30	KIF3B	20	kinesin family member 3B
	M30	MET	7	MET proto-oncogen receptor tyrosine kinase
	M30	PIP5K1B	9	phosphatidylinositol-4-phosphate 5-kinase type 1 beta
	M30	PRKAB1	12	protein kinase AMP-activated non-catalytic subunit beta 1
	M30	SPTBN1	2	spectrin beta non-erythrocytic 1
	M30	TJP2	9	tight junction protein 2
	M30	LDLR	19	low density lipoprotein receptor
	M30	TUBGCP2	10	tubulin gamma complex associated protein 2
	M30	RAN	12	RAN member RAS oncogene family
	M30	EPHB2	1	EPH receptor B2
	M30	ANK2	4	ankyrin 2
	M30	UBC	12	ubiquitin C
	M30	DYNLL2	17	dynein light chain LC8-type 2
	M9	TAC1	7	tachykinin precursor 1
	M9	TEAD3	6	TEA domain transcription factor 3
	M9	CLDN11	3	claudin 11
	M9	FLT4	5	fms related tyrosine kinase 4
	M9	COL11A1	1	collagen type XI alpha 1 chain
	M9	HDAC7	12	histone deacetylase 7
	M9	FGFR2	10	fibroblast growth factor receptor 2
	M9	CLTCL1	22	clathrin heavy chain like 1

	M9	ACTN1	14	actinin alpha 1
	M9	GLI2	2	GLI family zinc finger 2
	M9	NOTCH3	19	notch receptor 3
	M9	RASAL2	1	RAS protein activator like 2
	M9	ACTB	7	actin beta
	M9	IL4R	16	interleukin 4 receptor
	M9	COL16A1	1	collagen type XVI alpha 1 chain
	M9	MMP2	16	matrix metallopeptidase 2
	M9	NOS1	12	nitric oxide synthase 1
	M9	LAMB1	7	laminin subunit beta 1
	M9	MYL9	20	myosin light chain 9
	M9	FZD3	8	frizzled class receptor 3
	M9	TGFB1	19	transforming growth factor beta 1
	M9	USF2	19	upstream transcription factor 2 c-fos interacting
	M9	SCN1B	19	sodium voltage-gated channel beta subunit 1
	M9	GLI3	7	GLI family zinc finger 3
	M9	ACTA2	10	actin alpha 2 smooth muscle
	M9	COL1A1	17	collagen type I alpha 1 chain
	M9	MAP2K6	17	mitogen-activated protein kinase kinase 6
	M9	COL12A1	6	collagen type XII alpha 1 chain
	M9	LAMA4	6	laminin subunit alpha 4
	M9	RASGRF2	5	Ras protein specific guanine nucleotide releasing factor 2
	M9	PDGFRB	5	platelet derived growth factor receptor beta
	M9	COL7A1	3	collagen type VII alpha 1 chain
	M9	GNAI2	3	G protein subunit alpha i2
	M9	CBLB	3	Cbl proto-oncogene B
	M9	GNB4	3	G protein subunit beta 4
	M9	FN1	2	fibronectin 1
	M9	TGFB3	14	transforming growth factor beta 3
	M9	NPY	7	neuropeptide Y
	M9	CALD1	7	caldesmon 1

	M9	LRP1	12	LDL receptor related protein 1
	M9	COL10A1	6	collagen type X alpha 1 chain
	M9	PLCG1	20	phospholipase C gamma 1
	M9	C3	19	complement C3
	M9	NUP214	9	nucleoporin 214
	M9	SMO	7	smoothened frizzled class receptor
	M9	TNNI3	19	troponin I3 cardiac type
	M9	COL5A1	9	collagen type V alpha 1 chain
	M9	RAF1	3	Raf-1 proto-oncogene serine/threonine kinase
	M9	CHRM3	1	cholinergic receptor muscarinic 3
	M9	IRS4	X	insulin receptor substrate 4
	M9	COL4A2	13	collagen type IV alpha 2 chain
	M9	LAMC1	1	laminin subunit gamma 1
	M9	GABBR2	9	gamma-aminobutyric acid type B receptor subunit 2
	M9	TUBB2A	6	tubulin beta 2A class IIa
	M9	ITGA11	15	integrin subunit alpha 11
	M9	LEF1	4	lymphoid enhancer binding factor 1
	M9	COL2A1	12	collagen type II alpha 1 chain
	M9	ACVRL1	12	activin A receptor like type 1
	M9	CDH13	16	cadherin 13
	M9	GNAL	18	G protein subunit alpha L
	M9	COL6A1	21	collagen type VI alpha 1 chain
	M9	COL6A2	21	collagen type VI alpha 2 chain
	M9	HSPG2	1	heparan sulfate proteoglycan 2
	M9	TPM3	1	tropomyosin 3
	M9	COL8A1	3	collagen type VIII alpha 1 chain
	M9	SERPINH1	11	serpin family H member 1
	M9	MMP3	11	matrix metallopeptidase 3
	M9	ADAM17	2	ADAM metallopeptidase domain 17
	M9	GJA1	6	gap junction protein alpha 1
	M9	ASAP1	8	ArfGAP with SH3 domain ankyrin repeat and PH domain 1

	M9	MMP14	14	matrix metallopeptidase 14
	M9	FZD1	7	frizzled class receptor 1
	M9	SHC1	1	SHC adaptor protein 1
	M9	ITGA5	12	integrin subunit alpha 5
	M9	KCNJ3	2	potassium inwardly rectifying channel subfamily J member 3
	M9	COL6A3	2	collagen type VI alpha 3 chain
	M9	SPTA1	1	spectrin alpha erythrocytic 1
	M9	COL1A2	7	collagen type I alpha 2 chain
	M9	TUBA1A	12	tubulin alpha 1a
	M9	MLST8	16	MTOR associated protein LST8 homolog
	M9	RPSA	3	ribosomal protein SA
	M9	CTNNB1	3	catenin beta 1
	M9	COL3A1	2	collagen type III alpha 1 chain
	M9	AXIN2	17	axin 2
	M9	LIMS1	2	LIM zinc finger domain containing 1
	M9	PCDH7	4	protocadherin 7
	M9	CD14	5	CD14 molecule
	M9	FGG	4	fibrinogen gamma chain
	M9	COL8A2	1	collagen type VIII alpha 2 chain
	M9	LAMB2	3	laminin subunit beta 2
	M9	CEBPB	20	CCAAT enhancer binding protein beta
	M9	TUBB6	18	tubulin beta 6 class V
	M9	HLA-DQB1	CHR_HSCHR6_MHC_QBL_CTG1	major histocompatibility complex class II DQ beta 1
	M9	FZD2	17	frizzled class receptor 2
	M9	KCNH7	2	potassium voltage-gated channel subfamily H member 7
	M9	P4HB	17	prolyl 4-hydroxylase subunit beta
	M9	MYL6B	12	myosin light chain 6B
	M9	HLA-DQA1	CHR_HSCHR6_MHC_QBL_CTG1	major histocompatibility complexclass II DQ alpha 1
	M9	FLNA	X	filamin A
	M9	MAP3K5	6	mitogen-activated protein kinase kinase kinase 5

	M9	ITGBL1	13	integrin subunit beta like 1
	M9	COL5A2	2	collagen type V alpha 2 chain
	M9	NOTCH4	CHR_HSCHR6_MHC_QBL_CTG 1	notch receptor 4
	M9	ARPC4	3	actin related protein 2/3 complex subunit 4
	M9	UBE2V1	20	ubiquitin conjugating enzyme E2 V1
	M7	YWHAB	20	tyrosine 3-monooxygenase/tryptophan 5-monooxygenase activation protein beta
	M7	NRAS	1	NRAS proto-oncogene GTPase
	M7	HSP90AA1	14	heat shock protein 90 alpha family class A member 1
	M7	HMGB1	13	high mobility group box 1
	M15	BSG	19	basigin (Ok blood group)
**Common among DADA and WGCNA**	M30	CDON	11	cell adhesion associated oncogene regulated
	M30	WNT11	11	Wnt family member 11
	M30	DKK4	8	dickkopf WNT signaling pathway inhibitor 4
	M30	BMP2	20	bone morphogenetic protein 2
	M9	DKK3	11	dickkopf WNT signaling pathway inhibitor 3
	M9	GLI2	2	GLI family zinc finger 2
	M9	SMAD7	18	SMAD family member 7
	M9	TGFB1	19	transforming growth factor beta 1
	M9	GLI3	7	GLI family zinc finger 3
	M9	GLI1	12	GLI family zinc finger 1
	M9	DLL4	15	delta like canonical Notch ligand 4
	M9	MYC	8	MYC proto-oncogene bHLH transcription factor
	M9	MMP7	11	matrix metallopeptidase 7
	M9	SMAD4	18	SMAD family member 4
	M9	WNT7A	3	Wnt family member 7A
	M34	WNT5A	3	Wnt family member 5A

Table S10. Subtype classification in ST and LT PDAC survivors

Group	No	Subtype class	Correlation
ST	1	Desnoplastic	0.601720180657817
	2	Desnoplastic	0.570132634362687
	3	Desnoplastic	0.613362702709841
	4	Desnoplastic	0.617662291235527
	5	Desnoplastic	0.623144024983566
	6	Desnoplastic	0.612315218718657
	7	Desnoplastic	0.582184669712481
	8	Desnoplastic	0.607695854170024
	9	Stroma_Activated	0.656817489704527
	10	Desnoplastic	0.62689931758264
LT	1	Desnoplastic	0.639653762546883
	2	Pure_classical	0.650905066815171
	3	Immune_classical	0.573311509267934
	4	Desnoplastic	0.593265183325459
	5	Pure_classical	0.673809074302667
	6	Desnoplastic	0.647579081647793
	7	Pure_classical	0.578416897785757
	8	Desnoplastic	0.614192946616388
	9	Immune_classical	0.468034791127807

Fig. S1. A) Identification of biological markers: Summary of different level of analysis performed

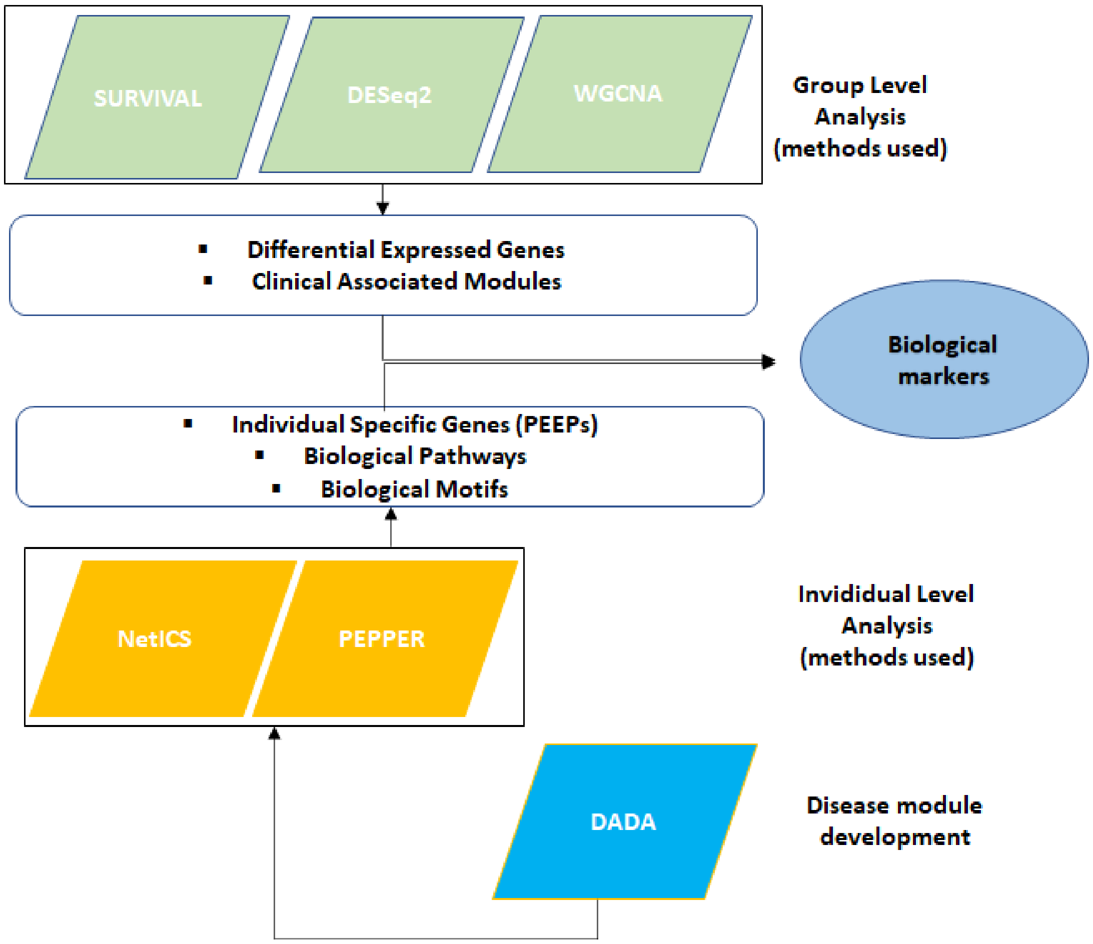

Fig. S1. B) CDD based top-ranked conserved domains in differentially expressed genes

Domain ID	Gene count	Domain Name
cl11960	16	Ig
cl29242	15	IG_like
cl29768	15	IG
smart00409	15	IG
smart00410	15	IG_like
pfam07686	13	V-set
cd00099	12	IgV
smart00406	12	IGv
cd04980	11	IgV_L_kappa
cd04982	11	IgV_TCR_gamma

Fig. S2. Kaplan-Meier plots for significant prognostic differentially expressed between long-term (LT: alive or death >=36 months) and short-term (ST: death >=3 months and <12 months) survival Gene miR-765, p-value < 0.0001. ST and LT group specific samples were classified into two subgroups i.e low and Mid based on normalized expression of miR-765 gene. The low and mid subgroup of ST are indicated with purple and blue while LT specific low and Mid subgroups are indicated with Green and Red color.

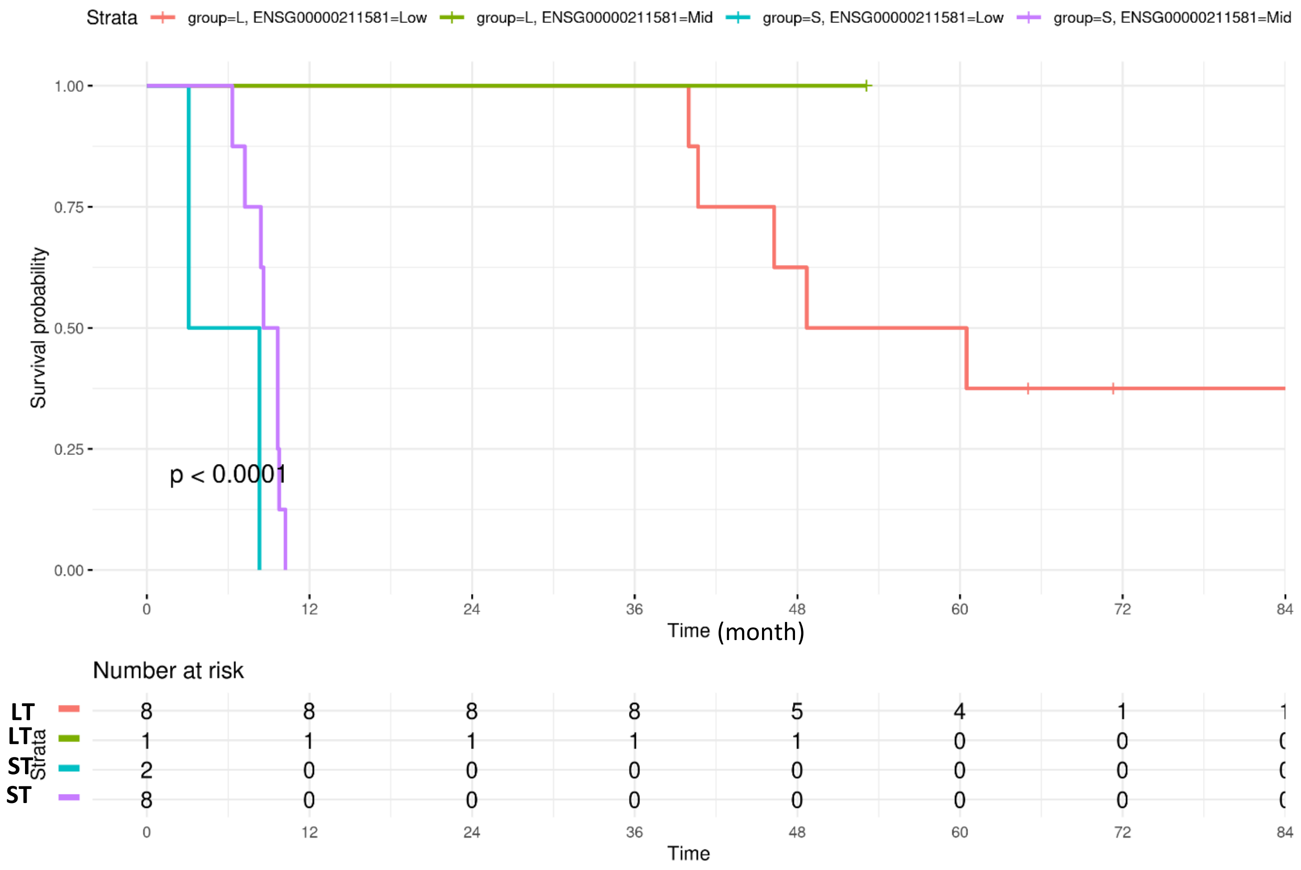

Fig S3. RNA-Seq supplemented with RT-PCR experiments: A. Correlation between RNA-Seq and RT-PCR expression values; B. Violin plot of TSPAN8 (gene ID: ENSG00000127324) by ST/LT PDAC survival; C. Same as B. but for REG4 (gene id: ENSG00000134193).

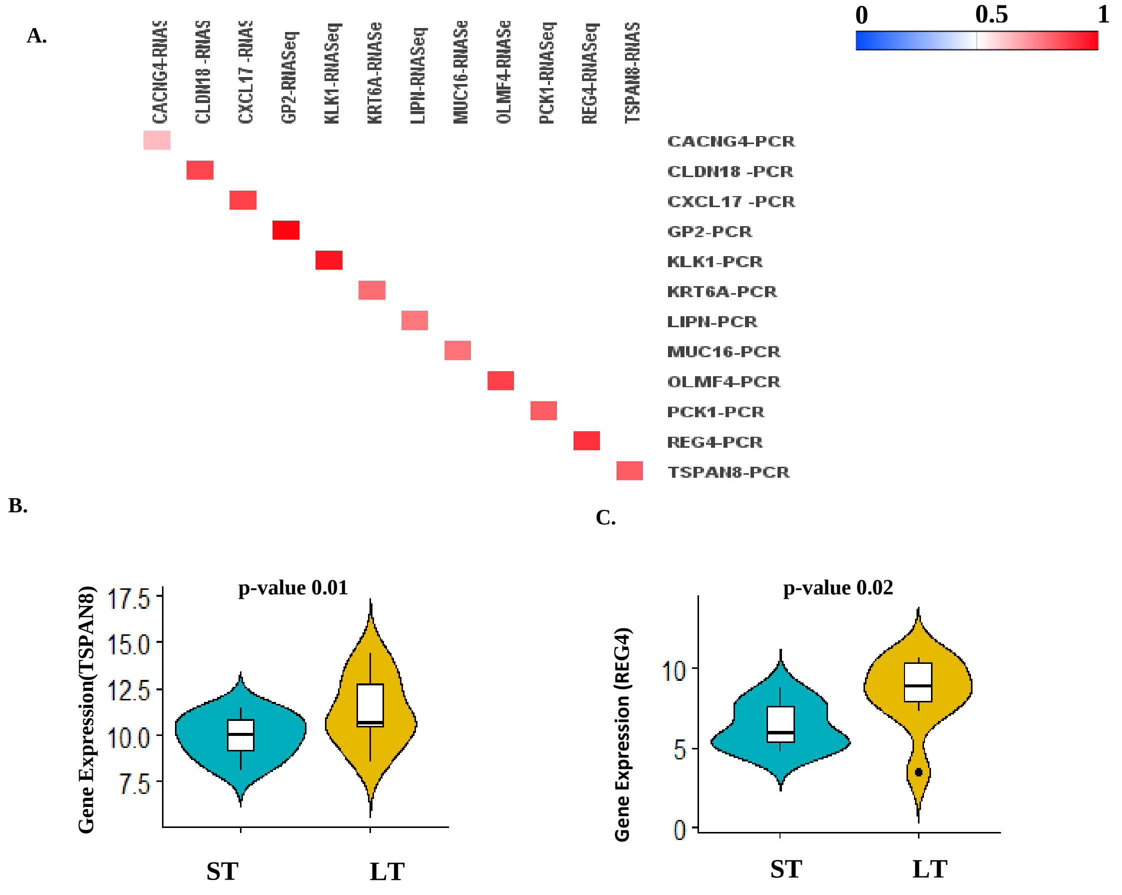

Fig S4. Clinical profile of WGCNA modules. Correlation patterns for WGCNA derived modules with significant association to clinical measurements (multiple testing adjusted p-value <0.05) are shown via corrplot[25] (supplementary method [section 2.1]). The more extreme the association (+1/-1) the deeper the color (dark blue/dark red). The sizes of the circles are proportional to the correlation coefficients. WGCNA = Co-Expression Network Analysis. Adjusted p values are not indicated in plot. Complete detail of significant modules is given in section: Survival group heterogeneity

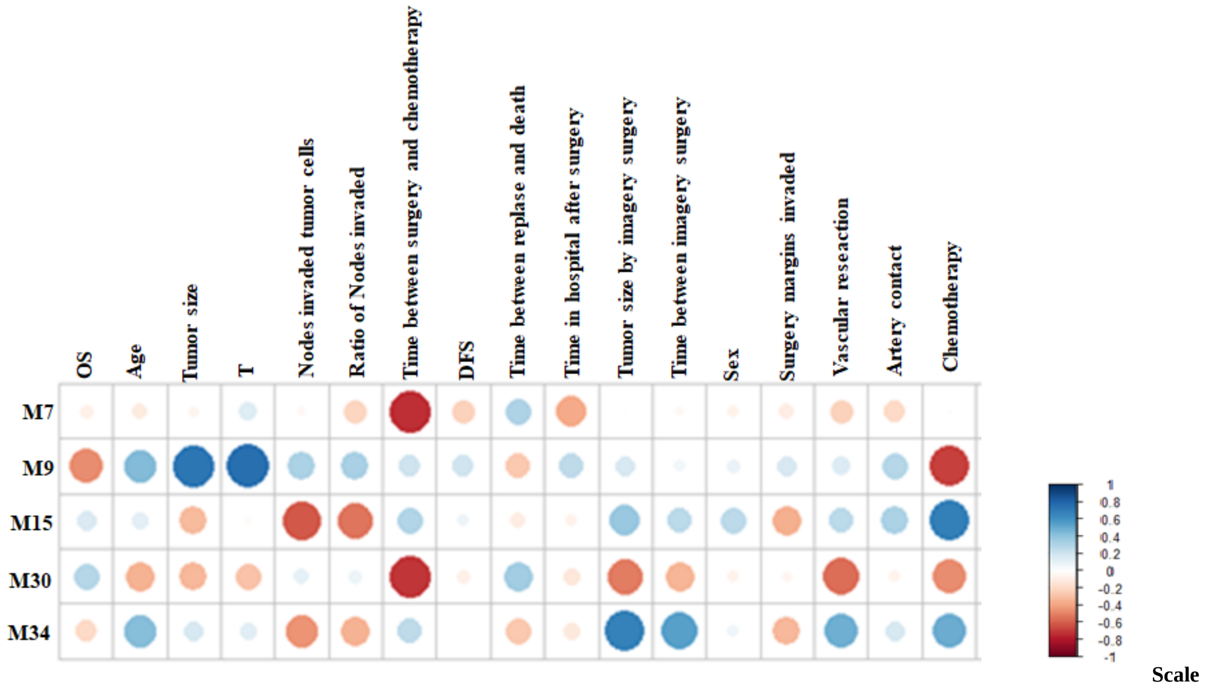

Fig. S5. Submap analysis in ST and LT PDAC survivors with PDAC subtype. Heatmap shows the association of ST and LT with known four PDAC subtypes. All four subtypes are represented as A1, A2 , A3 and A4 (A1 = ADEX; A2=Immunogenic , A3=Squamous and A4=Pancreatic Progenitor). Scale bar represents the pvalue identified from the SubMap module in GenePattern (https://www.genepattern.org/) method for the comparison of subtype specific cohort.

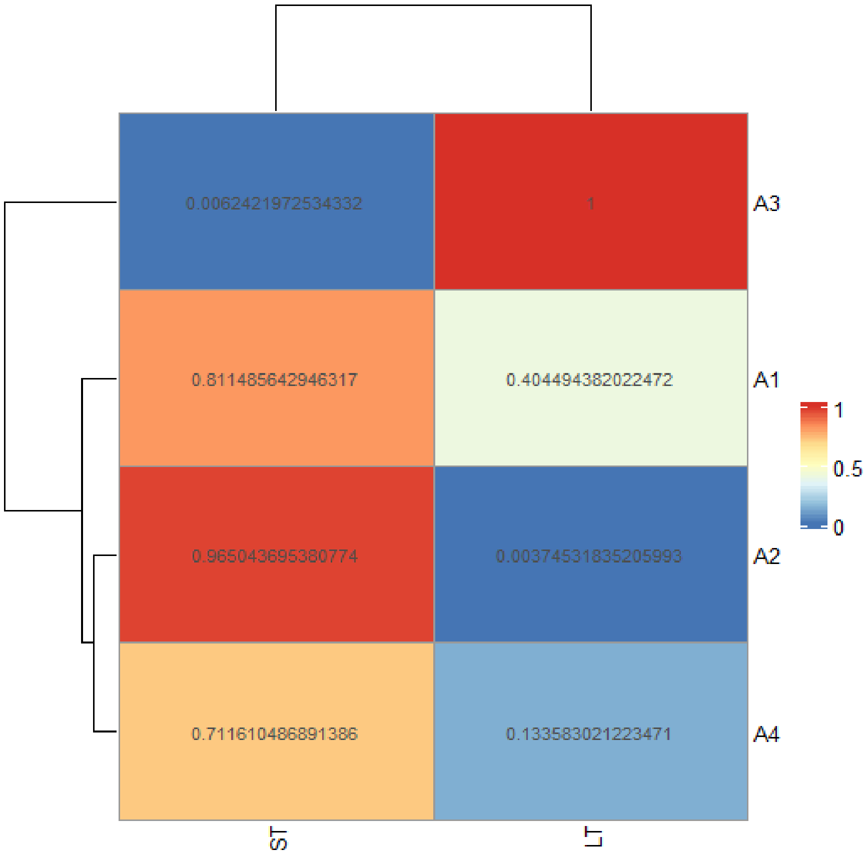

Fig. S6. Heatmap of identified PEEPs in LT PDAC survivors in three independent cohorts . Input matrix is logical with genes (not) perturbed in an individual indicated by 1 (0) in three independent cohorts A (I : collison et al 2011), B (Notta et al 2016) and C (TCGA). Red (1) and blue (0) color indicates the perturbation status of given gene in a corresponding sample.

I) II III

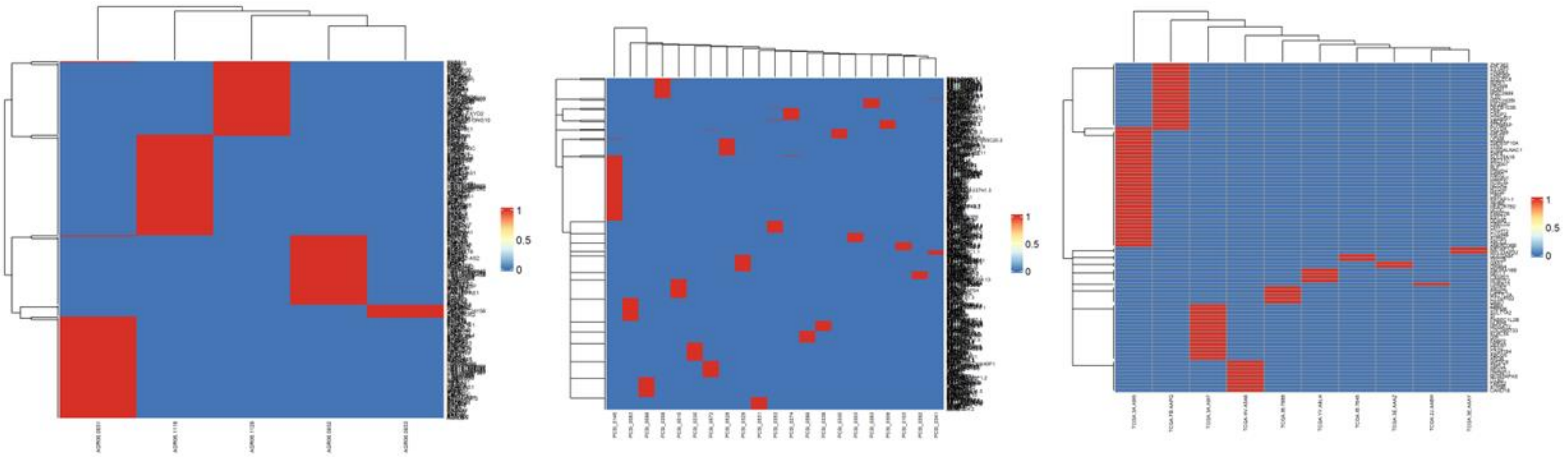

Fig. S7. Bicluster analysis of perturbed genes in LT PDAC survivors. Input matrix is logical with genes (not) perturbed in an individual indicated by 1 (0). A. Sixty-four biclusters (BC) obtained from such a logical matrix (section 2.2) indicated as heatmap. Each color in heatmap represent cluster number from 1 to 64. B.

Advanced interpretation of identified biclusters via three different approaches. (I) **bicluster membership graph** based on BC cluster x LTS. (II) **Heatmap based on Jaccard similarity index** computed for the identified 64 biclusters ranging from 0 (no concordance) to 1 (perfect concordance). (III) **Hierarchical tree** constructed for the identified biclusters (supplementary method [refer to section 2.2]). C. Functional annotation of 64 biclusters with clusterProfiler[6].

A.

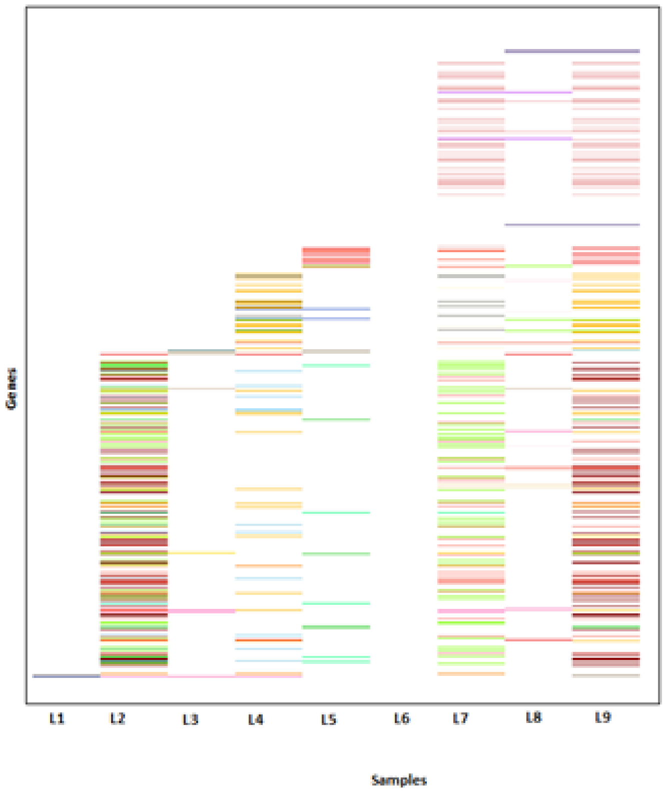

B.

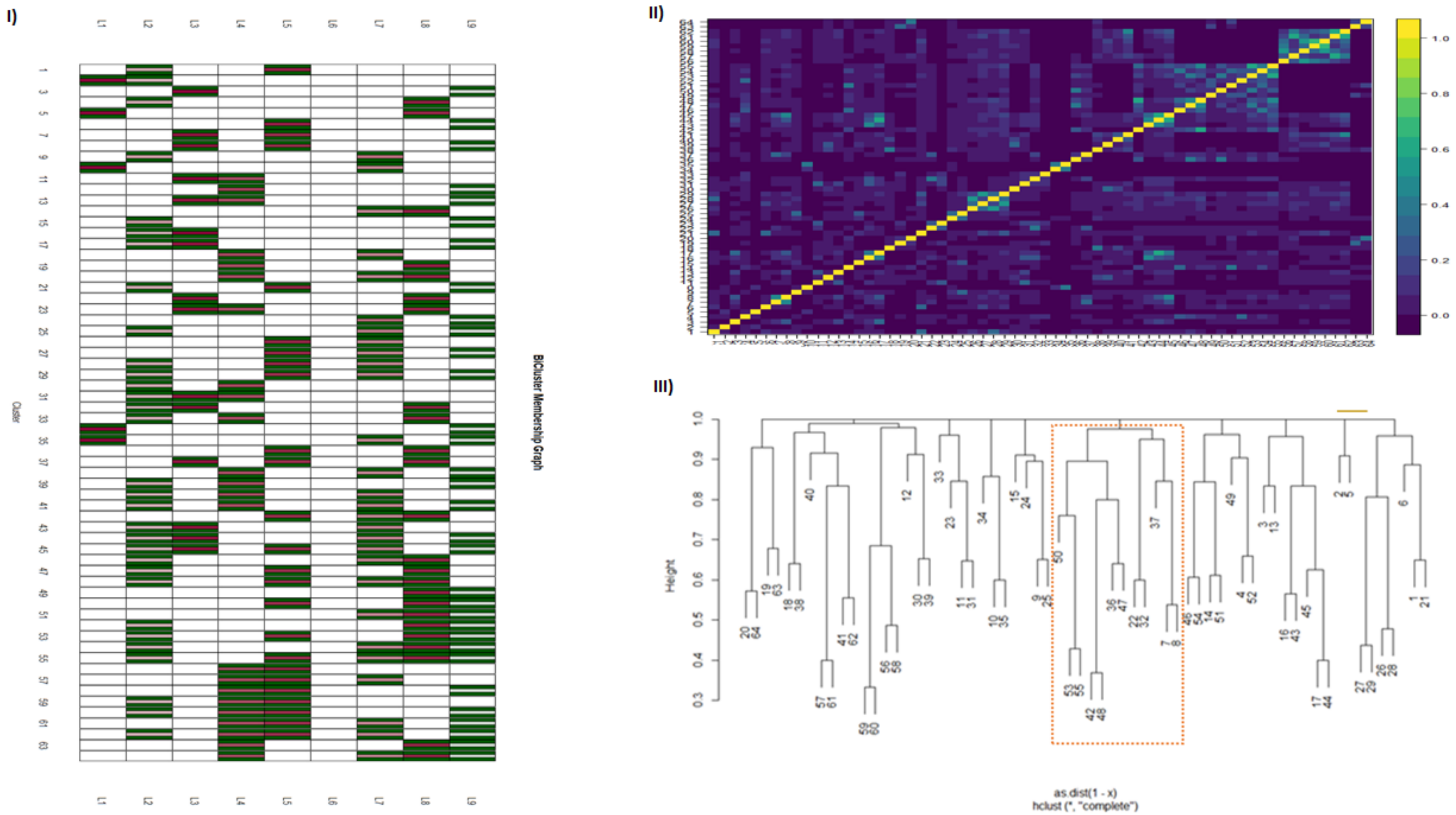
I)
II)
III)
BiCluster Membership Graph
Cluster
L1
L2
L3
L4
L5
L6
L7
L8
L9
Height
as.dist(1 - x)
hclust (*, "complete")

C.

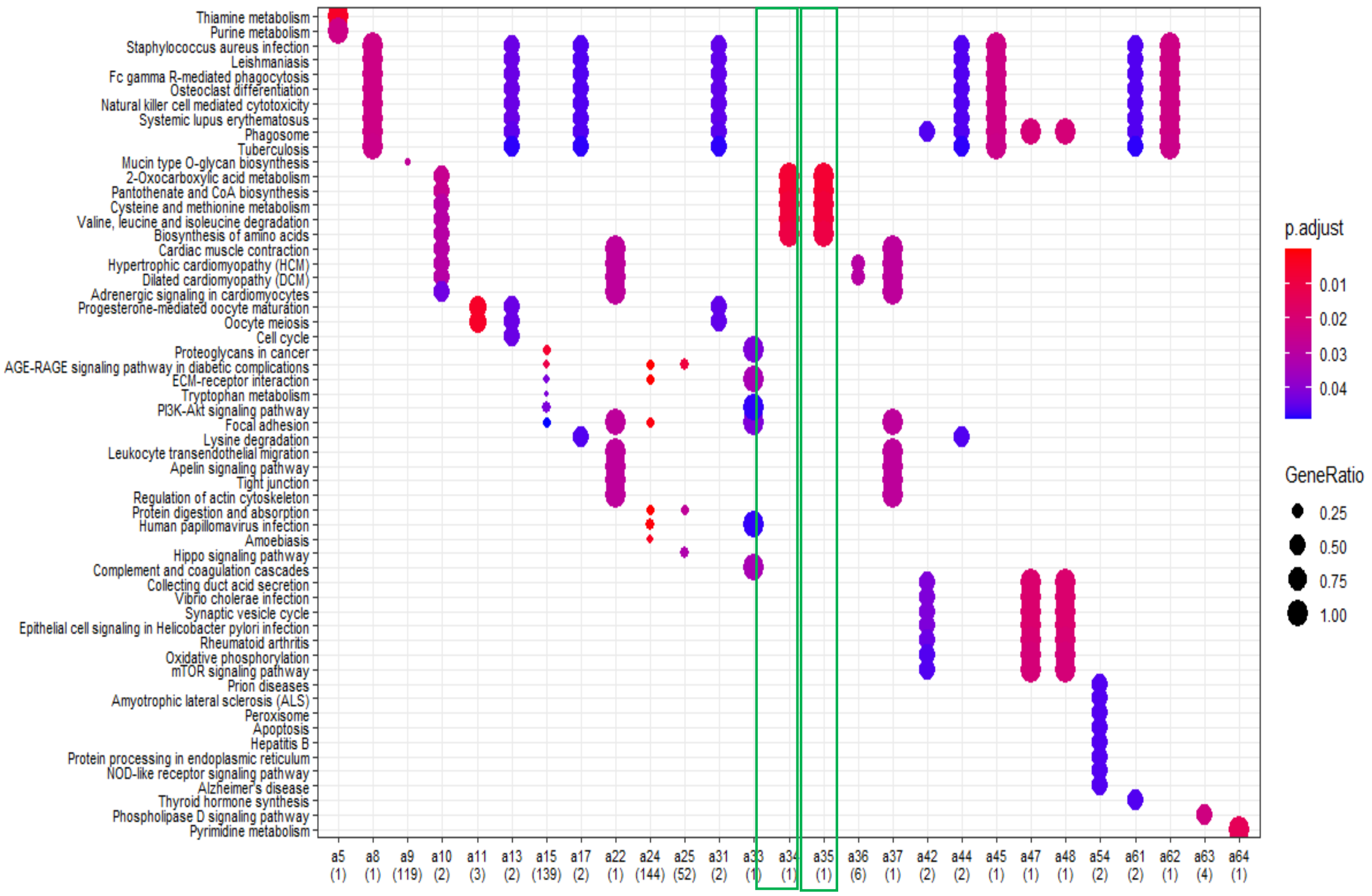

Fig. S8. Bicluster analysis of enriched pathways in LT PDAC survivors. Input matrix is logical with enriched pathways (not) in an individual indicated by 1 (0). A. Three biclusters (BC) obtained from such a logical matrix (section 2.2). B. Advanced interpretation of identified biclusters via three different approaches.

(I) **biclusters as lines of parallel coordinate graph** which indicates cluster specific detailed information. (II) **bicluster membership graph** based on BC cluster x LTS. (III) **Heatmap based on Jaccard similarity index** computed for the identified three biclusters ranging from 0 (no concordance) to 1 (perfect concordance). **Hierarchical tree** constructed for the identified biclusters (supplementary method [refer to section 2.2]).

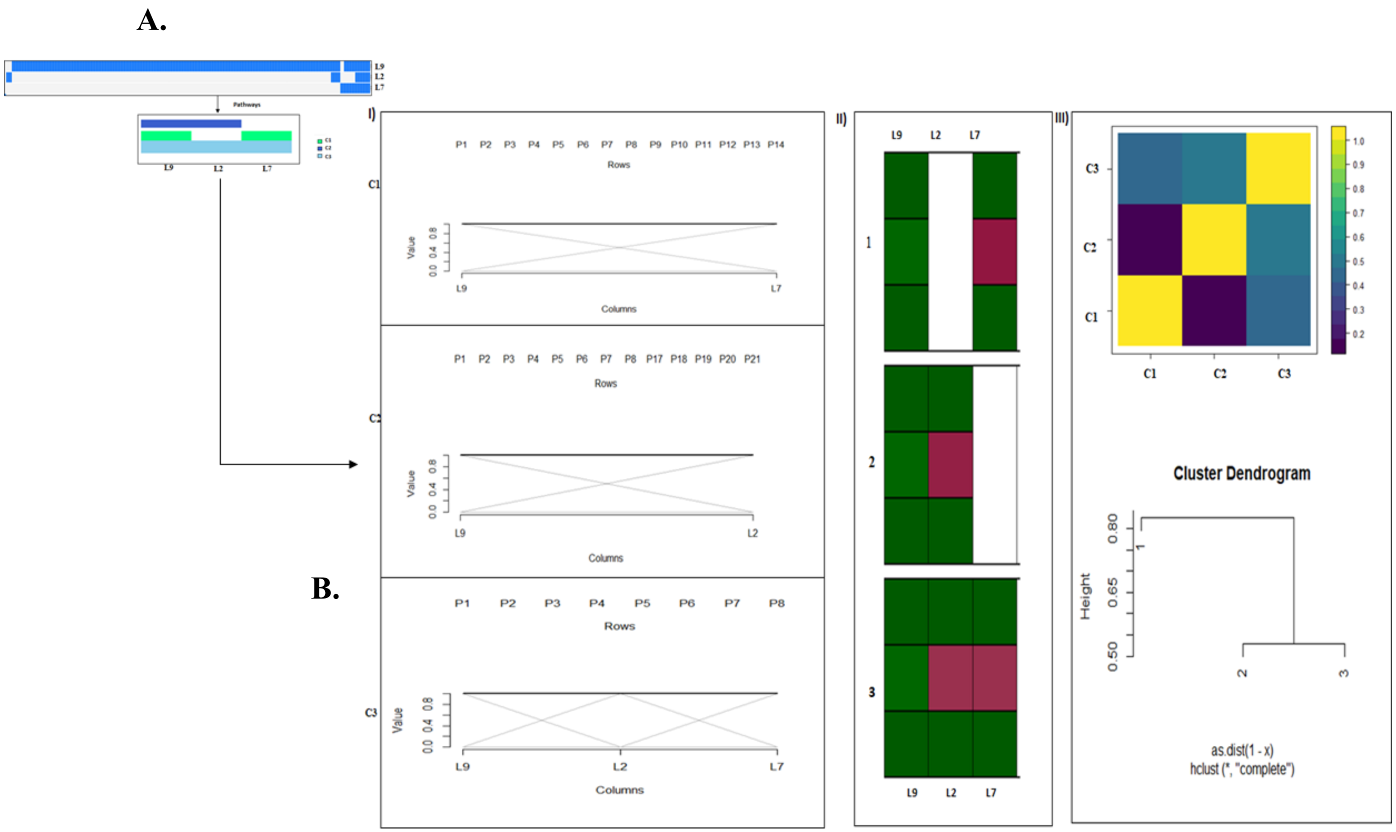

Fig. S9. Bicluster analysis of enriched protein domains in LT PDAC survivors. Input matrix is logical with enriched protein domains **(**not) in an individual indicated by 1 (0). A) Four biclusters (BC) obtained from such a logical matrix (section 2.2). B) Advanced interpretation of identified biclusters via three different approaches. (I) **biclusters as lines of parallel coordinate graph** which indicates cluster specific detailed information. (II) **bicluster membership graph** based on BC cluster x LTS. (III) **Heatmap based on Jaccard similarity index** computed for the identified 4 biclusters ranging from 0 (no concordance) to 1 (perfect concordance). **Hierarchical tree** constructed for the identified biclusters (supplementary methods).

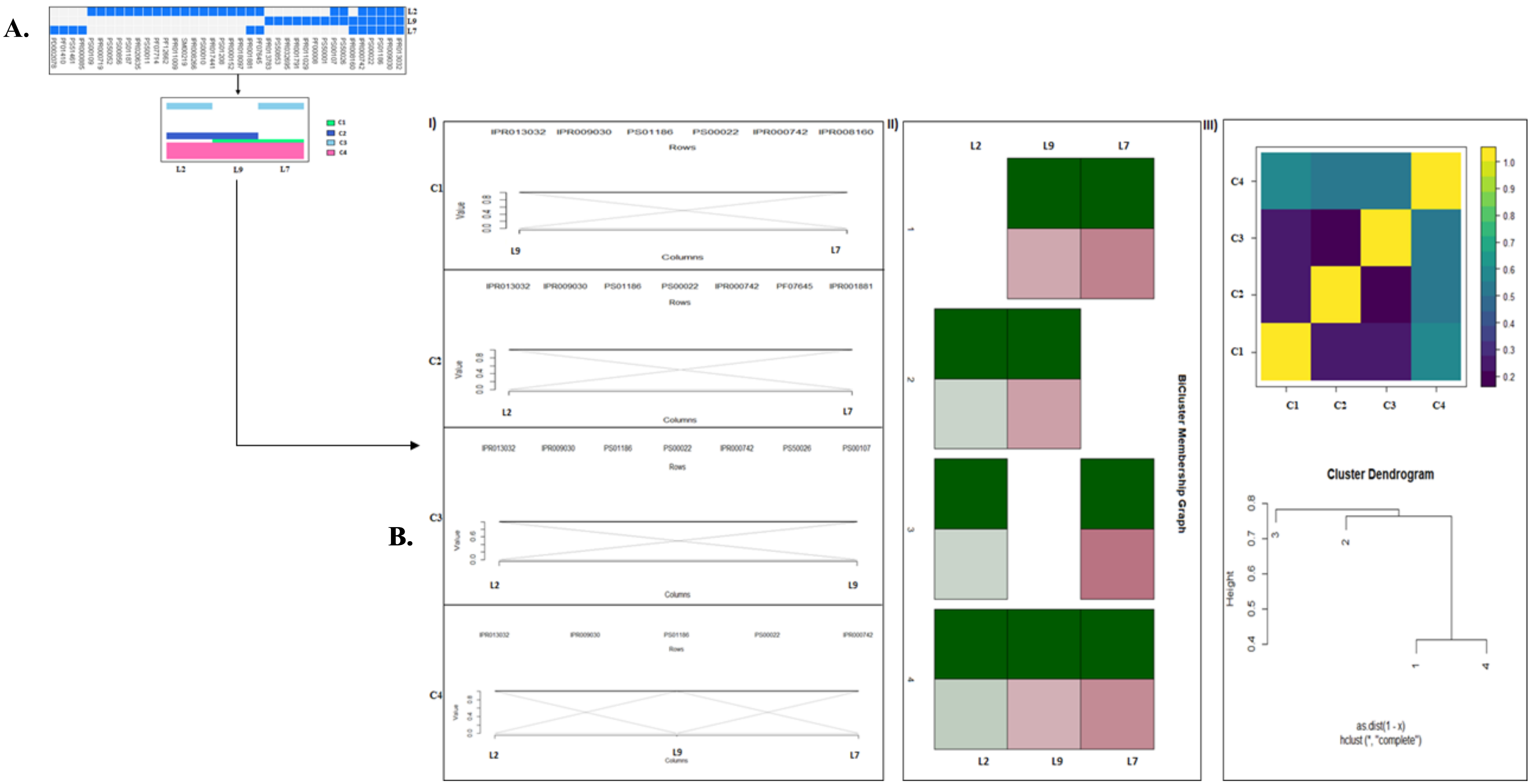

Fig. S10. Superposition of PEEP induced perturbed genes to PDAC disease module derived via DADA. Individual-specific perturbed genes as identified by individual profiling with PEPPER[15] (section 2.2) are highlighted with the same color per individual. LT2, LT3, LT4, LT5, LT7, and LT9 perturbed genes are indicated in red, green, light green, yellow, orange, and dark green, respectively. LT1, LT6 and LT8 specific perturbed genes showed no overlap in PDAC disease module derived via DADA (not shown in figure).

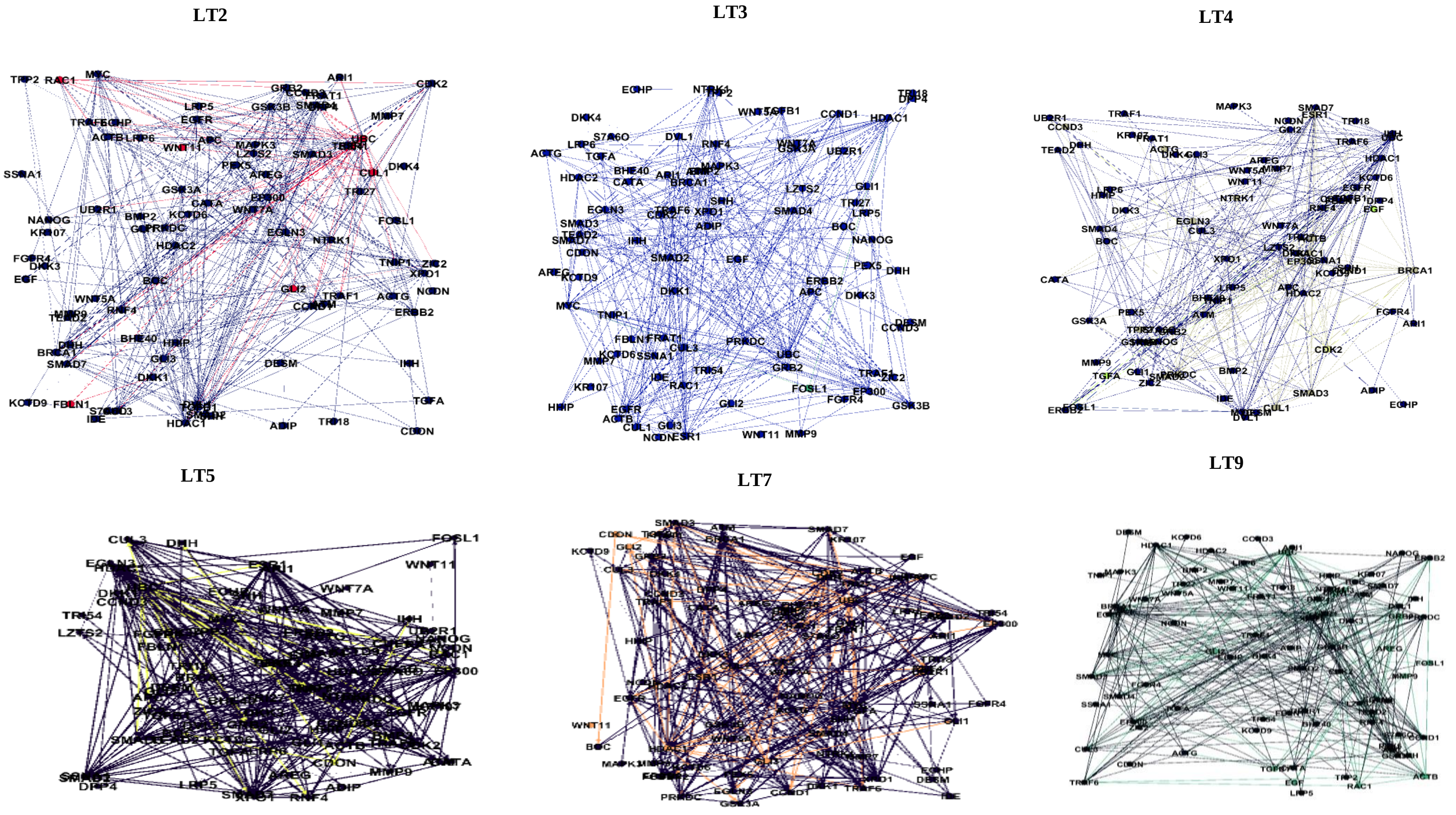

Fig. S11. Aggregation of multi-level analyses. Genes present in groups BC24 and BC25 (Figure S7) were shown to be involved in pathway sets C2 and C3 (Fig. S7) as indicated by the arrow.

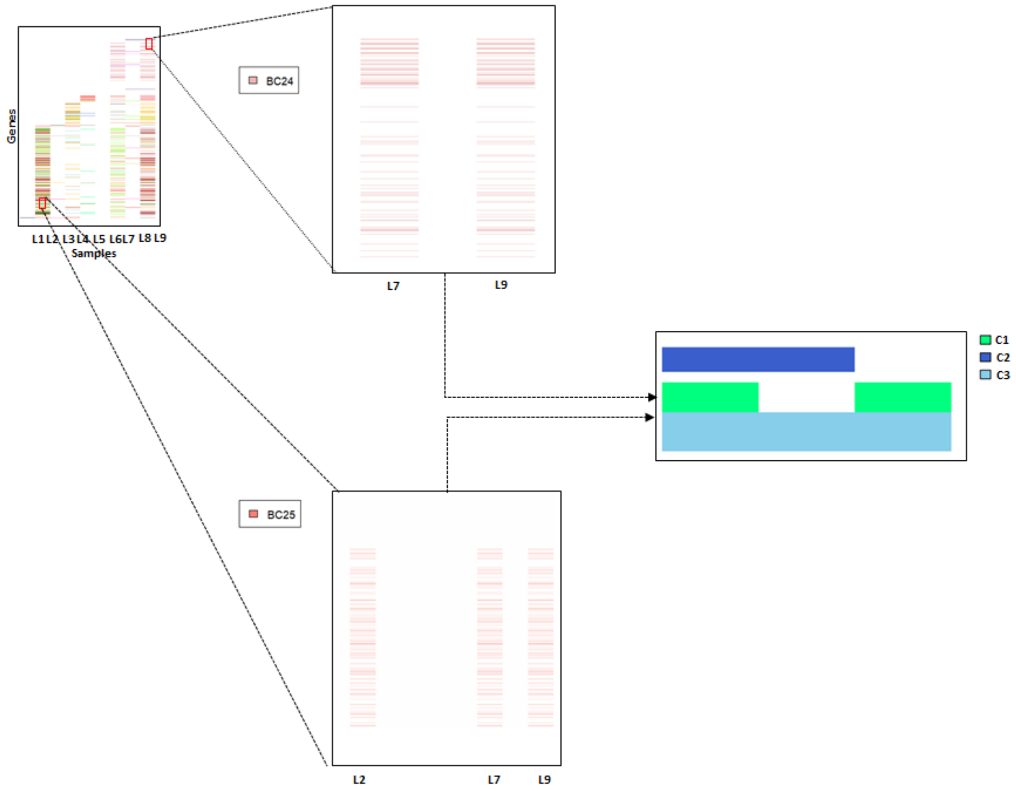

Supplement: Supplementary file 1 — Supplementary Information. [file 41598_2022_14592_MOESM1_ESM.pdf]
